# Actin- and Dynamin-Dependent Maturation of Bulk Endocytosis Restores Neurotransmission following Synaptic Depletion

**DOI:** 10.1371/journal.pone.0036913

**Published:** 2012-05-22

**Authors:** Tam H. Nguyen, Guillaume Maucort, Robert K. P. Sullivan, Mitja Schenning, Nickolas A. Lavidis, Adam McCluskey, Phillip J. Robinson, Frederic A. Meunier

**Affiliations:** 1 Queensland Brain Institute, The University of Queensland, Brisbane, Queensland, Australia; 2 School of Mathematics and Physics, The University of Queensland, Brisbane, Queensland, Australia; 3 Centre for Microscopy and Microanalysis, The University of Queensland, Brisbane, Queensland, Australia; 4 School of Biomedical Sciences, The University of Queensland, Brisbane, Queensland, Australia; 5 Chemistry, School of Environmental and Life Sciences, The University of Newcastle, Callaghan, Australia; 6 Cell Signalling Unit, Children's Medical Research Institute, The University of Sydney, Sydney, Australia; National Institutes of Health, United States of America

## Abstract

Bulk endocytosis contributes to the maintenance of neurotransmission at the amphibian neuromuscular junction by regenerating synaptic vesicles. How nerve terminals internalize adequate portions of the presynaptic membrane when bulk endocytosis is initiated before the end of a sustained stimulation is unknown. A maturation process, occurring at the end of the stimulation, is hypothesised to precisely restore the pools of synaptic vesicles. Using confocal time-lapse microscopy of FM1-43-labeled nerve terminals at the amphibian neuromuscular junction, we confirm that bulk endocytosis is initiated during a sustained tetanic stimulation and reveal that shortly after the end of the stimulation, nerve terminals undergo a maturation process. This includes a transient bulging of the plasma membrane, followed by the development of large intraterminal FM1-43-positive donut-like structures comprising large bulk membrane cisternae surrounded by recycling vesicles. The degree of bulging increased with stimulation frequency and the plasmalemma surface retrieved following the transient bulging correlated with the surface membrane internalized in bulk cisternae and recycling vesicles. Dyngo-4a, a potent dynamin inhibitor, did not block the initiation, but prevented the maturation of bulk endocytosis. In contrast, cytochalasin D, an inhibitor of actin polymerization, hindered both the initiation and maturation processes. Both inhibitors hampered the functional recovery of neurotransmission after synaptic depletion. Our data confirm that initiation of bulk endocytosis occurs during stimulation and demonstrates that a delayed maturation process controlled by actin and dynamin underpins the coupling between exocytosis and bulk endocytosis.

## Introduction

Neurotransmitter release relies on the exocytic fusion of synaptic vesicles with the presynaptic membrane at active zones [1]. In response to prolonged stimulation, synaptic vesicles are recycled by bulk endocytosis, which involves the internalization of large portions of the plasma membrane to form new synaptic vesicles [2], [3], [4] of the reserve pool [3], [5], [6], [7]. In most nerve terminals, bulk endocytosis has an activity threshold and is exclusively activated by high frequency stimulation [2], [8]. Bulk endocytosis is a general mechanism that occurs in nerve terminals of both the central nervous system as well as the peripheral nervous system [2], [3], [9], [10], [11]. A tight coupling between exocytosis and endocytosis ensures that the rate of synaptic vesicle formation equates with the rate of exocytic fusion. How nerve terminals achieve a precise coupling between exo- and endocytosis in situations when bulk endocytosis is triggered before the end of a long-term stimulation is unknown. This timing issue can be resolved by invoking a hypothetical maturation process that takes place after the end of the stimulation. This maturation would allow nerve terminals to precisely compensate for the number of synaptic vesicles that have undergone fusion during the entire time of the stimulation protocol. However, to the best of our knowledge, such a maturation process has not been described or hypothesized before.

Several lines of evidence implicate actin [12], [13], [14] and dynamin [14] as playing key roles in bulk endocytosis. Actin is involved in both endocytosis and exocytosis, and is associated with dynamic changes of the presynaptic plasma membrane [12]. At high frequency nerve stimulation, actin is involved in the recovery of the reserve pool of synaptic vesicles and sustaining synaptic transmission at the *Drosophila* [15] and frog [16] neuromuscular junction (NMJ), and in the giant reticulospinal synapse or the lamprey [17].

The importance of dynamin in endocytosis has been well established using organisms bearing defective dynamin such as the *Drosophila shibire* mutant [18] and mice deficient in dynamin-1 [19]. In both of these models, synaptic vesicle endocytosis is severely impaired and is characterized by the accumulation of numerous clathrin-coated pits and tubules lining the presynaptic membrane. In addition, the complete blockade of all forms synaptic vesicle endocytosis by the dynamin inhibitor, dynasore [14], under conditions of low and high frequency stimulation adds to the growing body of evidence suggesting a critical role for dynamin in bulk endocytosis. Thus, the mechanism coupling exocytosis and bulk endocytosis is likely to involve both actin and dynamin.

In this study we used the styryl dye FM1-43 and confocal time-lapse imaging at the amphibian NMJ in order to gain insight into the dynamic events taking place during bulk endocytosis. We show that activity-dependent bulk endocytosis is triggered at the onset of long-term high frequency stimulation and undergoes a maturation process that takes place after the end of the stimulation protocol. This maturation entails a transient nerve terminal bulging phase just preceding the appearance of large endosomes surrounded by recycling vesicles. We found a significant correlation between the amount of presynaptic membrane surface lost at the end of the bulging phase and the amount of membrane surface recovered in bulk endosomes and associated recycling vesicles, indicative of a tight coupling between exocytosis and bulk endocytosis. Finally, we found that both actin and dynamin play differentially important roles in this process.

## Results

### Bulk endocytosis is initiated during stimulation and undergoes a maturation process shortly after the end of stimulation

**Figure 1:**
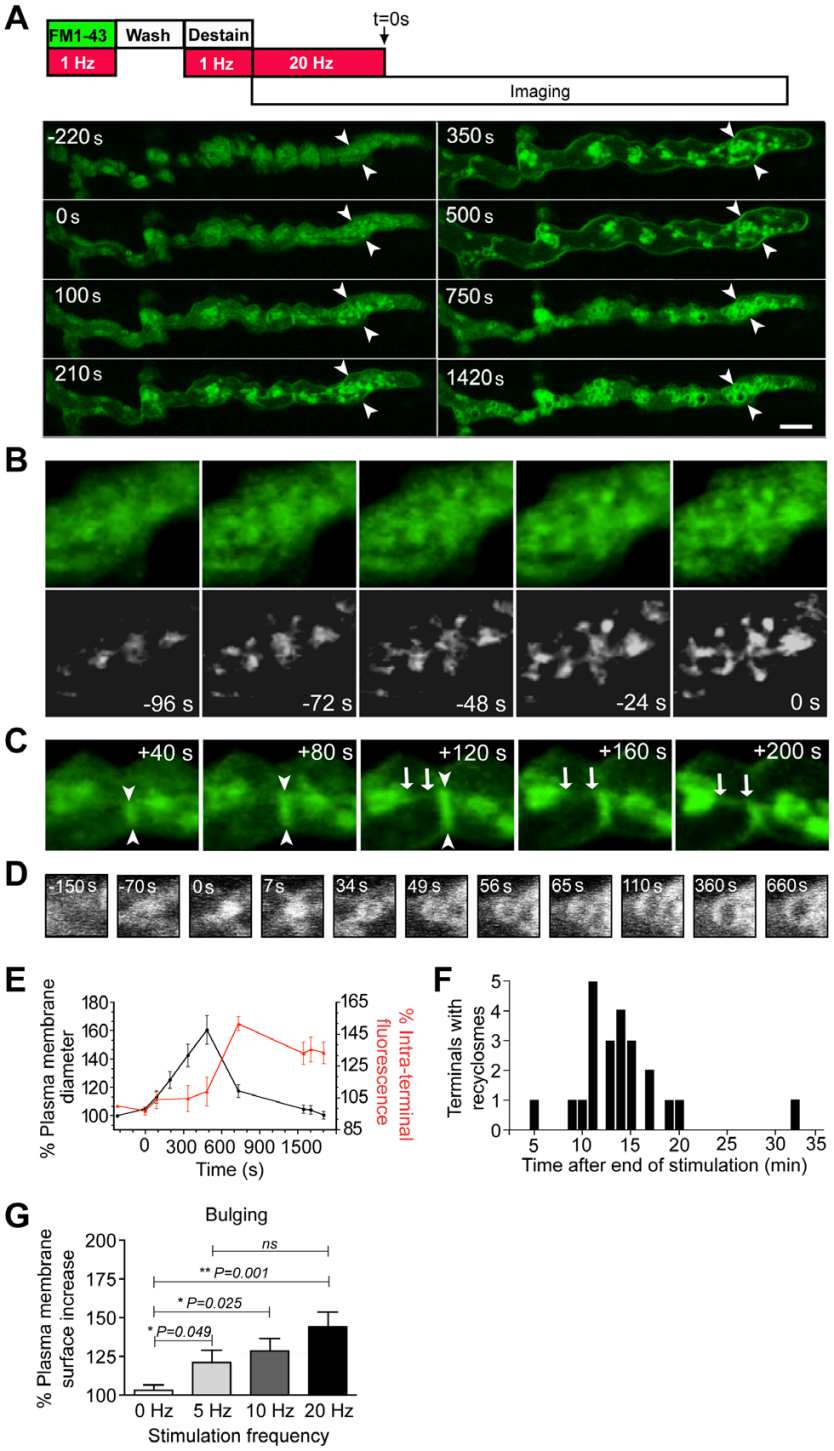
Bulging and collapsing of the presynaptic plasma membrane paves the way for bulk endocytosis. NMJ preparations were pre-labeled with FM1-43 by electrical stimulation (1 Hz for 5 min), and extensively washed in normal Ringer's solution. Labeled nerve terminals were then stimulated at 20 Hz for 10 min and visualized by time-lapse confocal imaging starting from the final 4 min of stimulation. The data shown are from a representative experiment, repeated independently 14 times. (**A**) Representative time-lapse confocal images of nerve terminals at various time points during and after stimulation (time = 0 sec indicates the end of the 20 Hz stimulation period). Note the various remodeling stages: the progressive increase in plasma membrane diameter (bulging, an example of a prominent section is indicated by arrowheads), the rapid increase in intraterminal fluorescence following the collapse in plasma membrane diameter, and the appearance of ‘bulk endosomes’. (**B** and **C**) Magnified regions of the same nerve terminal in (**A**) showing the initiation of bulk endosomes as FM1-43-positive balls interconnected by growing tubules either during (**B**) or after (**C**) the stimulation period (arrows and arrowheads indicate formation of FM-labeled tubules). Bottom panel in (**B**) shows over-contrasted versions of images in the top panel to improve signal-to-noise ratios. (**D**) Time-course of the increase in internal diameter of a representative bulk endosome during the maturation process. (**E**) Graph showing the percentage increase in plasma membrane diameter (black, n = 5) and percentage increase in intraterminal fluorescence (red, n = 8 region of interests) plotted over time. (**F**) Histogram of time taken for formation of recyclosomes in nerve terminals stimulated at 20 Hz. (**G**) Stimulation frequency dependency of nerve terminal bulging. Data shown as mean ± S.E.M and statistic significance was determined using Student's *t* test. Scale bar 5 µm.

In response to sustained levels of stimulation, motor nerve terminals exhaust their synaptic vesicles, leading to significant incorporation of synaptic vesicle membrane into the presynaptic plasma membrane. To investigate the mechanisms by which bulk endocytosis contributes to the regeneration of synaptic vesicle pools we performed time-lapse imaging of the toad *iliofibularis* NMJ stained with the styryl dye, FM1-43. FM1-43 has been used extensively to study synaptic vesicle recycling and is virtually non-fluorescent in solution and only becomes intensely fluorescent upon incorporation into membrane lipid compartments. In order to efficiently label the presynaptic plasma membrane, a 5 min pulse of FM1-43 was applied under 1 Hz stimulation to promote uptake of the FM dye into synaptic vesicles. The preparation was then washed and re-stimulated at 1 Hz for 5 min to promote “destaining” (FM1-43-labeled vesicles are anticipated to fuse with the plasma membrane as a result of exocytosis), resulting in the labeling of the presynaptic plasma membrane (Figure 1A). Motor nerve terminals were then stimulated at 20 Hz for 10 min to induce bulk endocytosis and time-lapse imaged during and after the end of the stimulation period (Figure 1A). It should be noted that although motor units have been shown to fire at rates of up to 50 Hz in an intermittent manner [20], sustained stimulation of nerve terminals at 20 Hz for 10 min is not strictly physiological and was used to augment the presence bulk endosomes. It addition, although the activity-dependent FM1-43 preloading and destaining of the nerve terminal to label the plasma membrane yielded a higher signal-to-noise ratio, it was not strictly necessary to visualize these events described as a 5 min passive FM1-43 staining of the presynaptic plasma membrane (dye binding to plasma membrane without stimulation) gave similar results (Figure S1). Intraterminal FM1-43-positive membrane balls interconnected by a network of tubules appeared shortly after the onset of stimulation (Figure 1B) as previously described [2], [10], and remained in the terminal long after the cessation of stimulation (Figure 1C). Importantly, following the end of stimulation, the nerve terminal underwent a striking series of dynamic events in the following order: (i) a bulging phase leading to a ∼60% increase in width and (ii) a rapid collapsing back to the initial diameter of the nerve terminal immediately followed by the appearance of large internal donut-like structures (Figure 1A and Movie S1). These donut-like structures are reminiscent of those found in motor nerve terminal stimulated with glycerotoxin [7], suggesting that they result from a maturation process of bulk endocytosis aimed at producing recycling vesicles. We thus refer to these structures as ‘recyclosomes’. To examine the formation of these recyclosomes, we followed the fate of the fluorescent balls and found that they undergo an ‘inflation’ process to reach the donut-shaped structure (Figure 1D), demonstrating that the fluorescent material originally present in the balls is translocated outwards as the large central cisternae develops. Quantitative analysis of time-lapse imaging experiments revealed a temporal coupling of nerve terminal bulging/collapsing, with the appearance of numerous intraterminal FM1-43-labeled structures always occurring during or shortly after the collapsing phase (Figure 1E–1F).

We next investigated the effect of varying stimulation frequency on the amount of nerve terminal bulging. Nerve terminals were stimulated at selected frequencies for 10 min, with FM1-43 being added during the last 5 min. In control, unstimulated conditions, FM1-43 staining was mainly located on the plasma membrane (Figure S2A) and no bulging was observed (Figure 1G). In nerve terminals stimulated at 5 Hz we could detect a degree of nerve terminal bulging that was statistically significant compared to control terminals (Figure 1G). Nerve terminals stimulated at 10 and 20 Hz exhibited higher degrees of bulging compared to control terminals (Figure 1G), with most of the FM1-43 associated with bulk endosomes (Figure S2B). These results indicate that the degree of nerve terminal bulging and recyclosome formation is dependent on stimulation-induced activity, particularly at higher frequencies.

Together, these results are consistent with bulk endocytosis being initiated soon after the onset of stimulation [2], [10] and a maturation process that takes place after the end of the stimulation, leading to the formation of bulk endosomes.

### Density of internalized membrane of bulk endosomes and surrounding recycled vesicles revealed by photoconversion

One possible function for the described maturation process could be to sense how much synaptic vesicle membrane is incorporated during the stimulation phase in order to adjust the amount of plasma membrane internalization required to replenish the pools of synaptic vesicles. To test this hypothesis, we carried out a series of experiments aimed at establishing a correlation between the amount of membrane lost during the collapsing phase and that internalized in bulk endosomes and surrounding recycling vesicles.

**Figure 2:**
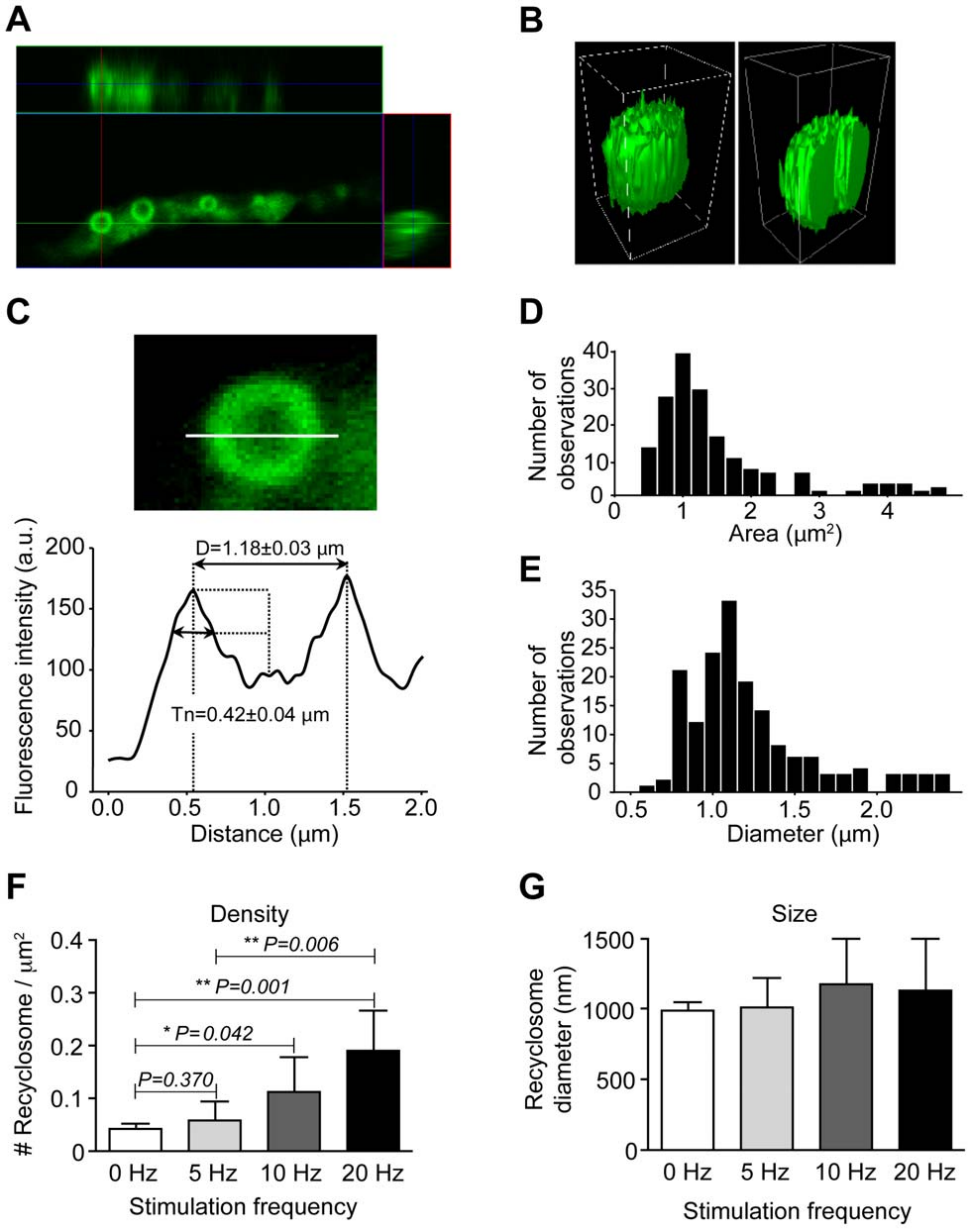
Morphometric characterization of donut-shape structures. Motor nerve terminals were electrically stimulated at 20 Hz for 10 min. FM1-43 (5 µM) was added 5 min before the end of the stimulation. The preparation was washed several times in normal Ringer's solution for 30 min, fixed using paraformaldehyde (4%), and rinsed in PBS. Confocal Z-stacks were acquired of nerve terminal branches containing donut-shape structures and two types of 3-dimensional representations are presented: (**A**) line scan and, (**B**) 3D surface rendering. (**C**) Confocal image of a donut-shaped structure and estimation of its diameter measured from the distance between the highest intensity taken from the fluorescence intensity profile as indicated. The average thickness was then calculated by establishing the size of the intensity profile taken equidistance between the center and the highest point of the intensity profile as indicated. Histograms showing the measured area (**D**) and diameter (**E**) of donut-shaped structures (n = 169 structures from 29 nerve terminals). Analysis of the recyclosome density (**F**) and recyclosome size (**G**) as a function of stimulation frequency. Data shown as mean ± S.E.M and statistic significance was determined using Student's *t* test.

To estimate the amount of membrane surface internalized in bulk endosomes and surrounding recycling vesicles we first investigated the 3-dimensional organization of the donut-like structures by confocal microscopy (Figure 2A–2B). The FM1-43 fluorescent halo surrounding the bulk endosome had an approximate thickness of 420 nm (Figure 2C), occupied an area ranging from 0.7 to 40 µm^2^ (Figure 2D) and had a diameter ranging from 0.6–2.5 µm (Figure 2E). Taking into account the point spread function of fluorescence, we expected that the recycling vesicles surrounding these bulky cisternae should therefore lie within ∼350 nm of the limiting membrane of the endosome. We have shown previously that the center of each donut-shaped structure is composed of a bulk endosome surrounded by an FM1-43-positive fluorescent ring comprising recycling vesicles [7]. These structures are similar in shape and size to those found under conditions of sustained stimulation promoted by glycerotoxin [7]. Fluorescence recovery after photobleaching (FRAP) experiments indicate that recyclosomes have lost most of their connection with the plasma membrane (Figure S3).

To estimate the amount of internalized membrane, we next measured the density of recyclosomes by confocal microscopy of nerve terminals stimulated at various frequencies. Image analysis revealed that their density was dependent on the stimulation frequency (Figure 2F). In the case of 20 Hz stimulation, recyclosomes occupied up to 10–15% of the total area of the nerve terminal, which corresponds to approximately 0.2 cisternae per µm^2^ of nerve terminal. (n = 340, 29 nerve terminals). The size and shape of the recyclosomes, however, remained the same at all stimulation frequencies (Figure 2G).

**Figure 3:**
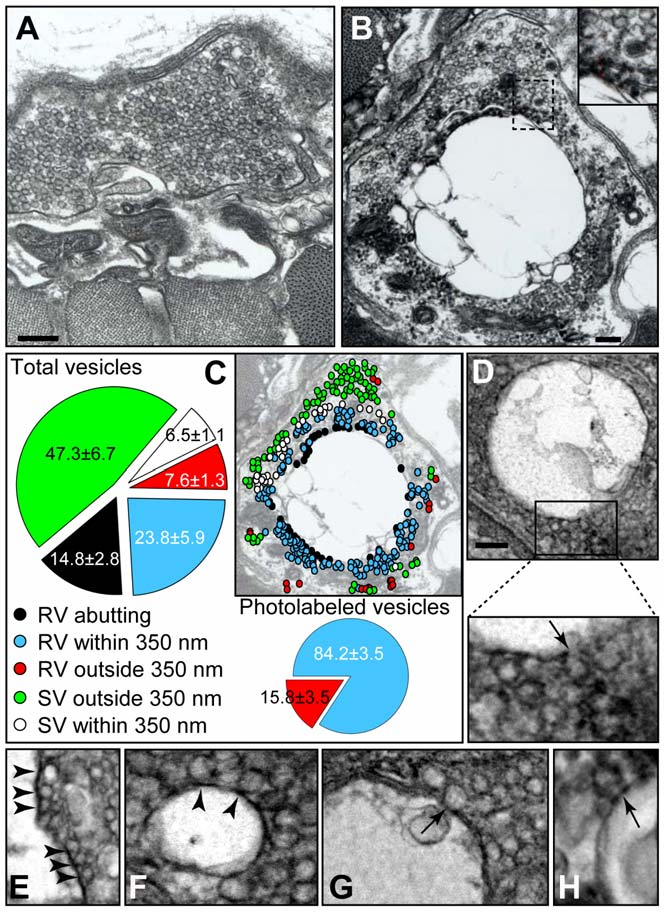
Estimation of the contribution of bulk endocytosis to synaptic vesicle recycling by photoconversion of FM1-43. Amphibian motor nerve terminals were stimulated at 20 Hz for 10 min and FM1-43 (5 µM) was added 5 min before the end of the stimulation. The preparation was then washed with Ca^2+^-free Ringer's solution, fixed and processed for photoconversion. (**A**) In unstimulated nerve endings, no apparent photoconversion was detected in the vesicular pool. (**B**) In stimulated nerve endings, large cisternae surrounded by photoconverted vesicles were observed. (**C**) Quantification of relative proportion of photoconverted vesicles. Vesicles were classified as recycling (RV; containing photoconverted electron-dense material) or synaptic (SV; non-photoconverted), falling into 3 categories: directly abutting the membrane of cisternae, within 350 nm of the cisternae and further away from the cisternae (color coding as indicated in the figure; n = 7 nerve terminals, 1580 vesicles quantified, 3 NMJ preparations). (**D**) Large intraterminal bulky structures and enlargement showing a few photolabeled vesicles in close contact with the membrane of the recyclosomes. The arrow indicates a probable nascent recycled vesicle. (**E–F**) Enlargement showing several photolabeled vesicles (arrowheads) in close contact with the membrane of the cisternae. (**G–H**) Enlargement showing photolabeled omega-shaped vesicles (arrow) in direct contact with the membrane of the cisternae. Scale bars 500 nm.

We next determined the amount of internalized membrane within the recycling vesicles surrounding the bulk endosomes by ultrastructural analysis of photoconverted FM1-43-stained preparations. Photoconversion of FM1-43-labeled NMJ preparations by exposure to blue laser light converts the dye into an electron dense stain that is detectable by electron microscopy. For estimation of recycling vesicle density, FM1-43-loaded NMJ preparations (20 Hz for 10 min) were photoconverted as previously described [21], [22], before being processed for electron microscopic analysis (Figure 3A and 3H). The majority of recycling vesicles (∼84%) were clustered in the immediate vicinity (within 350 nm) of the limiting membrane of the bulk cisternae (Figure 3B–3C). The remaining recycling vesicles (∼16%) found outside that zone were reminiscent of the photoconverted recycling vesicles previously described for milder stimulation protocols [23]. Closer examination of the cisternae revealed photolabeled vesicles in direct contact with the limiting membrane of the cisternae (Figure 3D and 3H). Notably, the limiting membrane of the bulk endosomes was often interrupted by omega-shaped structures, suggestive of vesicles budding off to form recycling vesicles (Figure 3D, 3F, and 3H). The size of the photoconverted vesicles varied but was generally smaller than that of synaptic vesicles (31.3±0.9 nm, n = 41). The volumetric density of photoconverted vesicles residing in the immediate vicinity of bulk endosomes was estimated by first determining the average number of photoconverted vesicles per µm^2^ within 350 nm of the bulk endosomal limiting membrane. A volumetric conversion factor was then applied (Equation 3, Materials and methods section) to derive a volumetric density of 17.23±2.28 vesicles per µm^3^.

### Bulging and collapsing of the presynaptic membrane couples exocytosis with bulk endocytosis

Based on our hypothesis, the amount of vesicular membrane incorporated into the presynaptic membrane during stimulation should be proportional to the amount of retrieved membrane in recyclosomes. In other words, the presynaptic membrane surface area lost during the collapsing phase should be proportional to the estimated amount of membrane internalized in both bulk endosomes and associated recycling vesicles. Amphibian nerve terminals are cylindrical in shape within a given region of interest (ROI), and we therefore assumed that the nerve terminal volume within the ROI could be approximated as a series of thin (one pixel thickness) cylinders (Figure S4A) from which the membrane surface area could be determined. The average volumetric density of recycling vesicles and their average diameter (Figure S4B and S4C) were estimated from electron microscopy sections of photoconverted NMJ preparations. Time-lapse microscopy of identified nerve terminals was used to estimate: (1) the surface area of presynaptic membrane lost during the collapsing phase, (2) the number of recyclosomes generated, (3) the thickness of their FM1-43-positive halos (Figure S4C), and (4) the membrane surface area of bulk endosomes.

**Figure 4:**
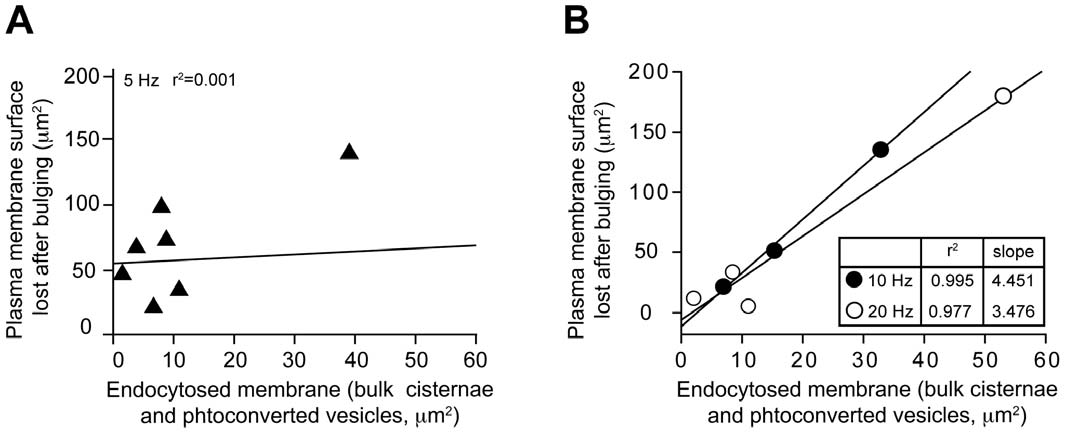
Bulging and collapse of nerve terminal plasma membrane couples exocytosis and bulk endocytosis. Confocal time-lapse imaging of nerve terminals stimulated at selected frequencies was used to determine the amount of membrane surface area lost during the collapse phase following 20 Hz stimulation. The amount of membrane internalized in bulk endosomes and associated recycling vesicles was determined by estimating the recycling vesicle density from ultrastructural analysis of photoconverted NMJ preparations. Regression analysis of the ratio between the membrane surface area lost during nerve terminal collapse and the amount of membrane endocytosed in bulk endosomes and surrounding recycling vesicles at 5 Hz (**A**) and 10–20 Hz (**B**).

For each nerve terminal, the plasma membrane surface area lost during the collapse was then compared to the membrane surface area endocytosed in bulk endosomes and surrounding recycling vesicles. Nerve terminals stimulated at 5 Hz did not exhibit a correlation between the lost plasma membrane and the internalized membrane surface (Figure 4A), suggesting that other types of endocytosis could be taking place at the same time and significantly contribute to membrane internalization. However, at higher frequencies (10 Hz and 20 Hz), a direct correlation between these two parameters could be detected, suggesting that at these frequencies, the collapse of the plasma membrane is indeed linked to the generation of endocytosed membrane (Figure 4B).

### Actin and dynamin act at different stages to induce activity-dependent bulk endocytosis

The pronounced dynamic changes to the presynaptic membrane observed in motor nerve terminals stimulated at 20 Hz, in addition to the demonstrated role of actin and dynamin in bulk endocytosis, suggested that both the actin cytoskeleton and dynamin might be involved in driving the maturation of bulk endocytosis. We therefore used the actin polymerization inhibitor cytochalasin D [24] and the dynamin inhibitor dyngo-4a [25] to assess the effect of actin and dynamin inhibition on the development of bulk endocytosis.

**Figure 5:**
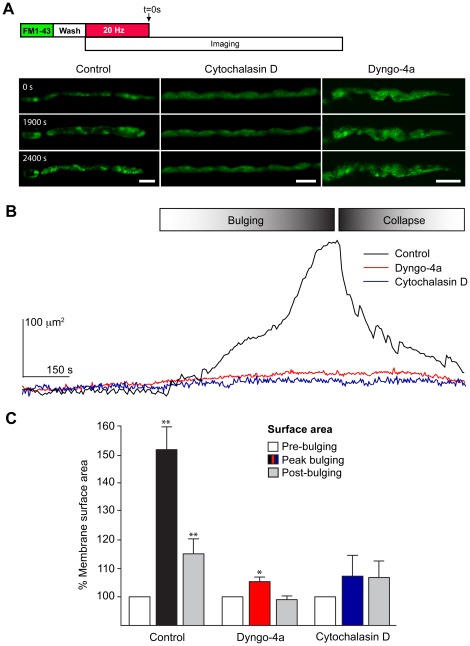
Disruption of actin polymerization and dynamin function inhibits plasma membrane bulging in nerve terminals stimulated at 20 Hz. NMJ preparations were pre-treated with cytochalasin D (4 µM) or dyngo-4a (30 µM) for 40 min followed by a 5 min pulse with FM1-43 (5 µM). The preparation was washed in the continuing presence of cytochalasin D or dyngo-4a prior to electrical stimulation at 20 Hz for 10 min and time-lapse imaging. The data shown are from representative experiments, repeated independently 3–14 times. (**A**) Untreated nerve terminals displayed a bulging of the plasma membrane followed by collapsing and formation of recyclosomes. Treatment of nerve terminals with cytochalasin D or dyngo-4a blocked the plasma membrane bulging and formation of recyclosomes. (**B**) Typical dynamics of changes in the plasma membrane surface area in untreated (black), cytochalasin D-treated (blue) or dyngo-4a-treated (red) nerve terminals. (**C**) Statistical analysis of the changes in the estimated surface area of presynaptic plasma membrane at the peak of bulging and after the collapsing phase. Untreated nerve terminals showed a clear bulging and collapsing in response to 20 Hz stimulation, whereas dyngo-4a-treated terminals showed a limited, but significant bulging in comparison. No detectable change was detected in cytochalasin D-treated nerve terminals. Data shown as mean ± S.E.M and statistical significance was determined using Student's *t* test. Scale bars 5 µm.

Motor nerve terminals were pretreated with cytochalasin D (4 µM) or dyngo-4a (30 µM) for 40 min, followed by a 5 min passive pulse of FM1-43 to pre-label the presynaptic plasma membrane. Preparations were then washed in the continuing presence of the appropriate inhibitor, and nerve terminals were visualized prior to and after stimulation at 20 Hz for 10 min. Time-lapse imaging showed that control untreated nerve terminals underwent a bulging and collapse process (Figure 5A–5C), as described earlier. Quantification of the change in nerve terminal surface area over time revealed that the bulging was a 2-phase process consisting of an initial slow phase followed by a faster phase, contributing 41.7±4.41 and 58.3±4.41% respectively, to the overall increase in nerve terminal surface area. In contrast, cytochalasin D not only inhibited the bulging of nerve terminals, but also the appearance of recyclosomes (Figure 5A–5C and Movie S2). Similarly, inhibition of dynamin by dyngo-4a also blocked the bulging of nerve terminals and the subsequent maturation of bulk endosomes (Figure 5A–5C), suggesting that dynamin is also involved in the bulging process. Although the effect of dyngo-4a in blocking the activity-dependent bulging was surprising, it is consistent with recently published data demonstrating that direct actin-dynamin interactions regulate the actin cytoskeleton [26].

### Dynamin inhibition blocks the maturation of bulk endosomes without interfering with the activity-dependent formation of membrane tubules

**Figure 6:**
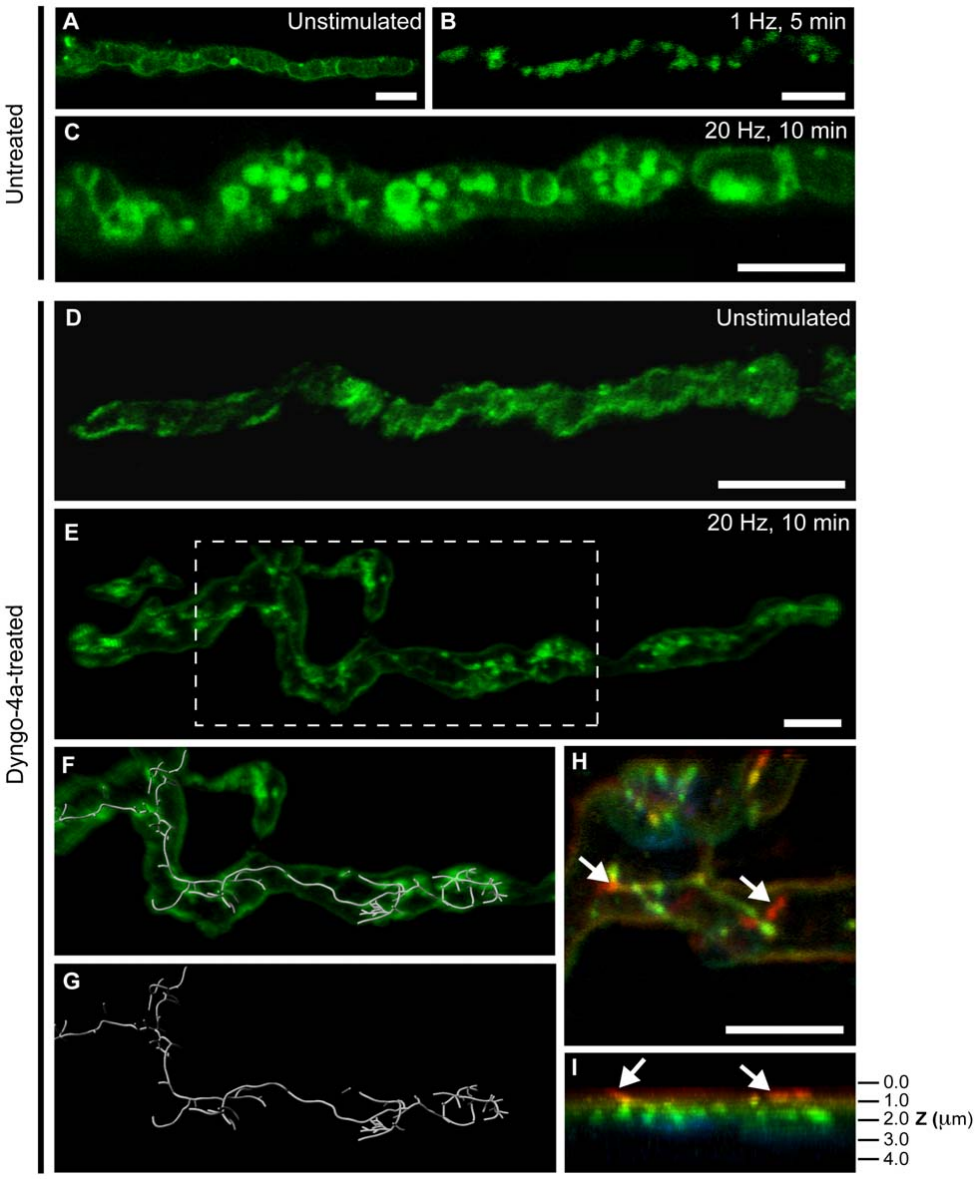
Dynamin inhibition blocks the formation of bulk endosomes and promotes intraterminal tubulation. NMJ preparations were treated with 30 µM dyngo-4a for 40 min in Ringer's solution (2 mM Ca^2+)^ followed by stimulation at 20 Hz for 10 min. A pulse of FM1-43 was applied for the last 5 min of stimulation, followed by washing with cold Ca^2+^-free Ringer's solution. Fresh Ringer's solution containing 30 µM dyngo-4a was then reapplied to the preparation, followed by confocal imaging. (**A**) Representative image of control unstimulated nerve terminal showing membrane localization of FM1-43. (**B**) Stimulation of untreated nerve terminals at low frequency (1 Hz) revealed the appearance of classical FM1-43-positive bands representative of synaptic vesicle clusters. (**C**) Stimulation of nerve terminals at 20 Hz revealed the appearance of large FM1-43-positive donut-shaped intraterminal structures. (**D**) Unstimulated dyngo-4a-treated nerve terminals exhibited similar plasma membrane FM1-43 staining to untreated terminals. Dyngo-4a-treated nerve terminals stimulated at 20 Hz exhibited a striking network of FM1-43-positive tubules that appeared to be connected to the plasma membrane (**E–I**). Filament trace modeling (**F** and **G**) demonstrates the reticulated nature of the branched tubular networks induced by dyngo-4a at the amphibian NMJ. (**H** and **I**), Depth coding analysis of nerve terminals revealed that the tubular networks were mostly located intraterminally, with most tubules residing within the green-yellow region (0.5 µm away from the plasma membrane). In some instances, the termination points of individual branches were located within the red region, indicating membrane localization (white arrows) of these branch terminals. Scale bars 5 µm.

We investigated the observed effect of dynamin inhibition on bulk endocytosis in greater detail by performing high-resolution confocal imaging of nerve terminals treated with dyngo-4a. When a 5 min passive pulse of FM1-43 (5 µM) was applied to NMJ preparations in the absence of electrical stimulation and FM1-43 staining was mainly localized to the plasma membrane (Figure 6A). The same procedure was then carried out with low frequency electrical nerve stimulation (1 Hz, 5 min) to promote the internalization of the styryl dye. After 5 min stimulation, the nerve terminals exhibited a regularly spaced pattern of labeling (Figure 6B) characteristic of the recycling vesicle organization previously reported at the frog NMJ [27]. Motor nerve terminals stimulated at 20 Hz for 10 min, with a pulse of FM1-43 applied during the last 5 min of stimulation, revealed the appearance of recyclosomes (Figure 6C), as previously described in Figure 1 [7]. The same experiments were carried out on NMJ preparations pre-treated with dyngo-4a (30 µM) for 40 min. Confocal imaging of unstimulated dyngo-4a-treated nerve terminals showed the expected weak FM1-43 labeling of the presynaptic plasma membrane and no significant internalization of the dye (Figure 6D). Dyngo-4a-treated 20 Hz-stimulated nerve terminals revealed a striking reticulated network of FM1-43-labeled tubular structures (Figure 6E–6G). Depth coding analysis, which assigns a color code to regions based on the depth of structures from a set point on the plasma membrane, showed that most of the FM1-43-labeled structures were located within the green-yellow region, indicating that these structures were extending well within the nerve terminal (Figure 6H–6I). There were clear instances of tubular branches that appeared to originate from the plasma membrane, as indicated by the red color-coding (Figure 6H–6I, white arrows), extending deep into the intraterminal space. Filament-trace modeling revealed that these reticulated fluorescent networks were intricate in nature (Figure 6F–6G and Movie S3). To confirm that these observed effects were specific to disruption of dynamin function, we tested other dynamin inhibitors, dynasore [28] and MitMAB [29]. Nerve terminals treated with either dynasore or MitMAB also prevented the formation of recyclosomes and resulted in the appearance of FM1-43-labeled structures seemingly connected with the nerve terminal plasma membrane (Figure S5A–S5D). Occasionally, some intraterminal tubules could be detected. Interestingly, a striated pattern of the FM1-43 staining was observed, and is suggestive of an accumulation of the dye in specific areas of the motor nerve terminal plasma membrane. Both these inhibitors led to an accumulation of FM1-43 on the plasmalemma, a blockade of recyclosome formation and the appearance of FM1-43-labeled intraterminal tubules (Figure S5A and D arrowhead).

Ultrastructural analysis of dyngo-4a-treated nerve terminals labeled with the fluid-phase marker, horseradish peroxidase (HRP), confirmed findings of the FM1-43 imaging experiments. Stimulated nerve terminals displayed numerous small-diameter tubular membrane structures (Figure S6B–S6G). Interestingly, most of the HRP accumulation was detected in tubules that were proximal to the plasma membrane (Figure S6C), confirming that at least some of these tubules were indeed connected to the plasma membrane. This suggests that dyngo-4a perturbs the maturation of bulk endosomes at the level of the plasma membrane by preventing the pinching off of newly formed bulk endosomes. Nerve terminals treated with dynasore (80 µM), another dynamin inhibitor [14], [28], also prevented the formation of bulk endocytosis (data not shown).

### Dyngo-4a inhibits dynamin-1 function but not its recruitment to the plasma membrane

**Figure 7:**
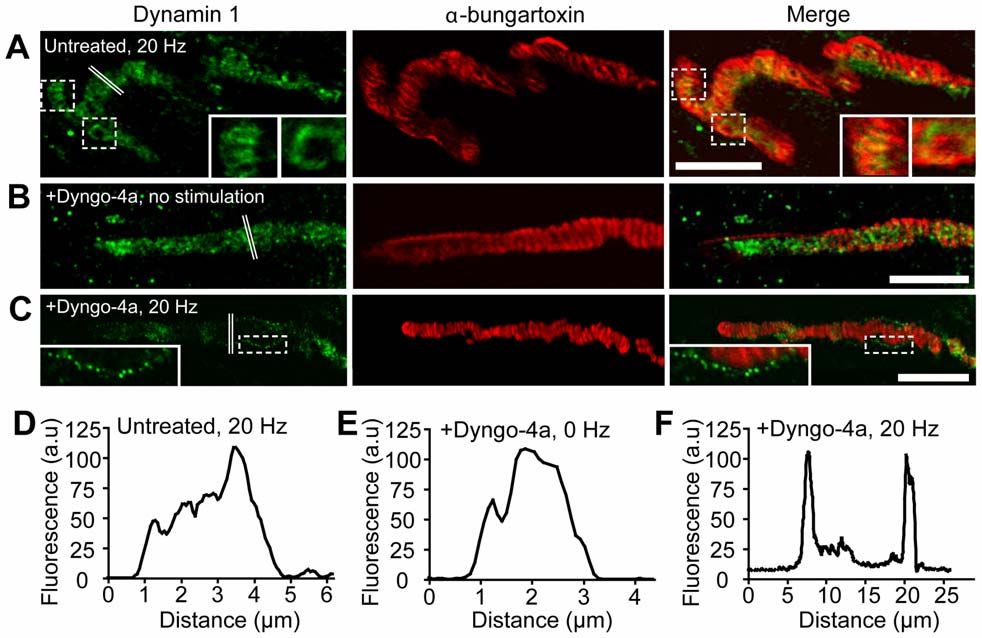
Dyngo-4a inhibits dynamin-1 function but not its recruitment to the plasma membrane. NMJ preparations were stimulated at 20 Hz for 10 min in either the presence or absence of dyngo-4a, followed by fixation with paraformaldehyde and immunostaining for dynamin-1. (**A**) Stimulation of nerve terminals not treated with dyngo-4a demonstrated that dynamin is localized to bulk endosomes, as well as active zones as revealed by α-bungarotoxin labeling of postsynaptic acetylcholine receptors (insets). (**B**) Control unstimulated nerve terminals treated with dyngo-4a show dynamin-1 distribution throughout the nerve terminal. In contrast, immunostaining of stimulated nerve terminals treated with dyngo-4a (**C**) revealed dynamin-1 staining localized to hotspots lining the plasma membrane, similar to the FM1-43-labeled hotspots seen in the early stages of bulk endosome formation (Figure 4). (**D–F**), Line intensity profile analysis on selected regions (double white lines) was performed to determine the fluorescence distribution. Scale bars 5 µm.

The blockade of bulk endosome maturation and the tubulating effect of dyngo-4a prompted us to investigate the possible change in dynamin-1 distribution under stimulation in the presence or absence of dyngo-4a. In untreated 20 Hz-stimulated nerve terminals, dynamin-1 was localized intraterminally in both large donut-shaped structures (reminiscent of recyclosomes) and α-bungarotoxin-positive bands (Figure 7A (see inserts) and 7D). In unstimulated nerve terminals treated with dyngo-4a, dynamin-1 had a punctate intraterminal distribution and no obvious colocalization with α-bungarotoxin-positive bands (Figure 7B and 7E). In sharp contrast, dynamin-1 staining was significantly altered in 20 Hz-stimulated dyngo-4a-treated nerve terminals (Figure 7C and 7F). Most dynamin-1 was clustered in hotspots of the plasma membrane, with comparatively little intraterminal staining. Our data therefore suggest that dynamin-1 is in dynamic equilibrium between the plasma membrane and intraterminal structures. This raises the possibility that, upon stimulation, dynamin-1 transiently translocates to the plasma membrane before being cycled in bulk endosomes and recycling vesicles. Dyngo-4a could therefore act by blocking dynamin-1 redistribution at the level of the plasma membrane. Our data further indicate that dyngo-4a acts downstream of dynamin-1 recruitment to the plasma membrane to prevent the fission of large portions of the plasma membrane destined for bulk endocytosis and recycling vesicles.

### Dynamin and actin inhibition blocks the functional recovery of neurotransmission from synaptic depletion

We next investigated the functional consequences of blocking bulk endocytosis and its maturation using dynamin and actin polymerization inhibitors. We first checked whether dyngo-4a had an effect on phasic quantal neurotransmitter (action potential-evoked synchronous) release [30]. NMJ preparations were initially treated with 30 µM dyngo-4a for 40 min prior to stimulation at 0.5 Hz and endplate potential (EPP) amplitudes were recorded for a period of 3 min (Figure S7A–7B). Surprisingly however, we did not detect any significant change in EPP amplitude. We therefore increased the dyngo-4a dosage to 100 µM and recorded EPP amplitudes for 30 min. Under these conditions, the EPP amplitude of untreated preparations remained stable over the time course of our experiment (Figure S7C–7D), whereas dyngo-4a had a biphasic action, including an initial slight potentiation followed by a mild rundown (Figure S7E–7F). This result is consistent with that found using dynasore, another dynamin inhibitor [31].

**Figure 8:**
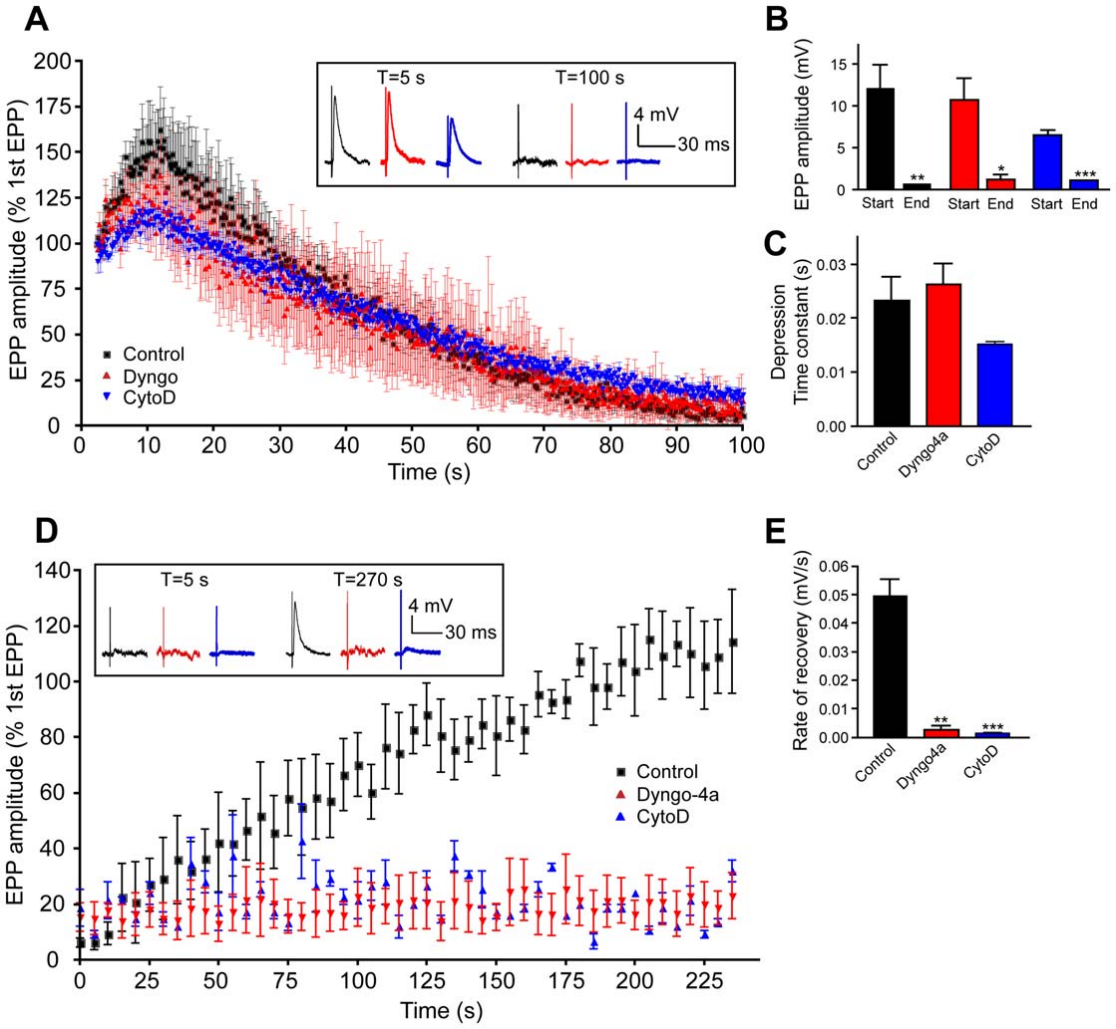
Dyngo-4a and cytochalasin D inhibit recovery from synaptic depression at the neuromuscular junction. NMJ preparations were either incubated with dyngo-4a (30 µM) or cytochalasin D (4 µM) or left untreated for 40 min in normal Ringer's solution. Synaptic depression was induced by stimulation at 20 Hz for 10 min. (**A**) Time-course of mean amplitude of EPPs (expressed as percentage of the average of the first 10 EPPs) from 4 terminals during stimulation elicited at 20 Hz in control (black), dyngo-4a-treated (red) and cytochalasin D-treated (blue) preparations as indicated. The inset in (**A**) shows representative EPP traces at the indicated time points. (**B**) Comparison of the averaged EPP amplitudes at the indicated time and conditions. (**C**) Comparison of average time constants of the exponential fits performed on each synaptic depletion time-course in the indicated conditions. (n = 4). (**D–F**), EPPs were elicited by electrical stimulation at a frequency of 0.5 Hz following 20 Hz high-frequency depletion to monitor recovery of neurotransmission. (**D**) Time-course of mean EPP amplitudes from 3 terminals during stimulation elicited at 0.5 Hz in control, dyngo-4a-treated and cytochalasin D-treated preparations as indicated. The inset in (**D**) shows representative EPP traces at the indicated time points. (**E**) Comparison of the rate of recovery of neurotransmission from synaptic depletion for the indicated conditions. (n = 3). Data shown as mean ± S.E.M and statistical significance was determined using Student's *t* test.

To probe whether the contribution of dynamin and actin function during high-frequency stimulation requires the replenishment of both the reserve pool (RP) and the readily-releasable pool (RRP), a tetanic train of 20 Hz electrical stimulation, a procedure known to exhaust most synaptic vesicle pools [32], [33], was applied to NMJ preparations either in the absence or presence of dyngo-4a or cytochalasin D respectively. In untreated preparations, a transient facilitation was detected, followed by a slow run down of the EPP amplitude culminating in a complete depression of phasic release – a process known as synaptic depletion (Figure 8A–8C). Importantly, both dyngo-4a and cytochalasin D had no significant effect on the rate of synaptic depression (Figure 8A–8C), suggesting that dynamin and actin inhibition does not readily impact on phasic release, at least during protocols of synaptic depletion. Following synaptic depression, the return of evoked neurotransmitter release can be followed by low-frequency stimulation (0.5 Hz). Under these conditions, the functional return of neurotransmitter release takes several minutes (Figure 8D–8E). Importantly, in the presence of either dyngo-4a or cytochalasin D, the recovery of phasic neurotransmitter release was completely abolished (Figure 8D–8E). Consequently, the rate of recovery of phasic release was significantly reduced in preparations treated with either dyngo-4a or cytochalasin D when compared to untreated preparations (Figure 8E). These results demonstrate that dyngo-4a and cytochalasin D dramatically inhibit the functional recovery of quantal phasic synaptic transmission following synaptic depletion at the amphibian NMJ.

## Discussion

In this paper, we examined the dynamic events underpinning the initiation and subsequent maturation of activity-dependent bulk endocytosis at the amphibian NMJ. Our results confirm that initiation of bulk endocytosis takes place during stimulation [2]. Importantly, we provide evidence that a delayed form of maturation of bulk endocytosis supports the coupling between exo- and bulk endocytosis – a process differentially controlled by actin and dynamin.

Our data demonstrate that bulk endocytosis is triggered shortly after the onset of stimulation, a finding that is consistent with previous studies performed in the central nervous system: cerebellar granule neuronal cultures [2] and the calyx of Held [34]. Our stimulation protocol, aimed at depleting synaptic vesicles, was stronger than that used in these previous studies. However, our results confirm that internalization of FM1-43-labeled presynaptic plasma membrane occurs during such stimulation and is initiated through the formation of FM1-43 hotspots on the presynaptic membrane that evolve into fluorescent balls and tubules. We found that this network of internalized membrane continued to grow after the cessation of stimulation, a process consistent with a form of maturation of the bulk endosomal compartment that has not been previously described. This maturation process might be triggered by the longer duration of our stimulation (20 Hz for 10 min) intended to deplete both the RRP and RP of synaptic vesicles [32]. Recently, similar donut-like structures were detected in presynaptic nerve terminals treated with the stimulatory neurotoxin glycerotoxin [7].

Our study provides quantitative analysis of the percentage of recycling (photoconverted) vesicles associated with bulk endosomes. The size and shape of the bulk cisternae and the halo of fluorescence surrounding the limiting membrane of the endosomes, further supported by our photoconversion EM analysis, suggest that recycling vesicles still associated with plasmalemmal invaginations (fluorescent balls) closely follow the maturation of invagination into bona fide bulk endosomes. The majority of recycling vesicles (∼84%) were clustered in the immediate vicinity of the bulk endosomes. Other recycling vesicles (∼16%) found outside that zone were reminiscent of the photoconverted vesicles previously described [23]. Whether the omega-shaped structures detected represent fusion or fission of recycling vesicles with the cisternae is unknown and is beyond the scope of this study. It is worth noting that due to the high density of small recycling vesicles surrounding each bulk endosome, the actual percentage of internalized membrane found on bulk endosomes was negligible (<1%) when compared to that of recycling vesicles.

The observed significant correlation between the amount of bulging/collapsing and internalized membrane surface at high-frequency stimulation indicates that this form of plasticity is essential to prepare the plasma membrane for the development of bulk endocytosis and points to a sensing role of the bulging phase in determining how much vesicular membrane was incorporated into the presynaptic membrane during the exocytic phase. The increase in membrane surface area expected to result from high levels of exocytic fusion should be paralleled with a decrease in cytoplasmic volume due to the loss of volume previously occupied by synaptic vesicles [35]. In conditions of high stimulation, an increase of nerve terminal surface area has previously been described in bipolar nerve terminals [36]. This was linked to the increase in plasma membrane surface area resulting from the exocytic fusion of synaptic vesicles. To the best of our knowledge, our study is the first to describe changes in nerve terminal volume associated with activity. At this stage, we can only invoke the possibility of a compensatory mechanism such as an inward hydrodynamic flow to balance the reduced cytoplasmic volume perhaps associated with dynamic changes in the cytoskeleton. More work is required to assess these possibilities.

The lack of correlation between the amount of bulging/collapsing and internalized membrane surface observed at 5 Hz stimulation suggests that at this frequency, other forms of endocytosis such as clathrin-mediated single vesicle recycling may also be contributing to the regeneration of synaptic vesicles pools. At the amphibian NMJ, a switch from single vesicle to bulk endosomal synaptic vesicle recycling has been reported [37]. Our findings are consistent with such a switch in the mode of endocytosis, from single vesicle to bulk endosomal, occurring at higher frequency stimulation.

Our data point to a critical role of actin and dynamin during the maturation of bulk endocytosis. Our results show that the kinetics of presynaptic bulging exhibits two distinct phases: a slow period followed by a fast phase reaching a plateau just preceding the collapse. Together with molecular motors such as myosins [12], [38], actin has previously been reported to act in tension sensing [39], [40]. It is tempting to speculate that, at the plateau, the actomyosin machinery involved in bulging has reached a point at which the membrane resistance opposes its forces. Actin-binding proteins promoting fast actin depolymerization [41] could be involved in the collapsing phase. The parallel internalization, formation of bulk endosomes and generation of surrounding recycling vesicles is likely to involve polymerization and branching of actin as previously proposed [15], [16], [17], perhaps via a tread-milling effect [42].

Our data are in good agreement with previous studies [37], [43] regarding the role of actin polymerization in the recovery of neurotransmitter release after synaptic depletion at the NMJ. The slow nature of functional recovery further suggests that actin driven bulk endocytosis is primarily responsible for replenishing the reserve pool of synaptic vesicles as previously proposed [6], [37], [43].

Dynamin inhibition completely blocked bulk endocytosis under high frequency stimulation conditions. The detection of branched tubulation networks in nerve terminals treated with various dynamin inhibitors and the altered distribution of dynamin-1 immunoreactivity in dyngo-4a-treated stimulated nerve terminals argue for an effect of dynamin on the maturation of bulk endocytosis. A key assumption of this hypothesis is that the observed tubules are still connected with the plasma membrane, a situation supported by our confocal imaging and electron microscopy data. The molecular mechanisms underpinning the formation of these branched tubules following dynamin inhibition are unknown. However, BAR domain proteins, such as amphiphysin and endophilin, could be involved in the early membrane-deforming stages of endocytosis [44], [45]. Furthermore, BAR proteins have been shown to be part of an actin regulatory network and, upon overexpression, to induce tubular invagination of the plasma membrane [46]. Perhaps not surprisingly, several studies have demonstrated both a direct interaction between dynamin and actin [26] and an indirect interaction via proteins that bind both dynamin and actin [47], [48], [49]. Moreover, there is increasing evidence for a functional link between endocytosis and actin dynamics, with actin being involved in specific steps of the endocytic process [50], [51]. In view of the role of dynamin in regulating the actin cytoskeleton [52], [53], [54], disruption of dynamin function and subsequent dysregulation of actin function are consistent with the formation of extensive tubulation of the plasma membrane observed in our imaging experiments. Our results are also in good agreement with the electron microscopy data generated with the dynamin-1 knockout mouse [19] and with the temperature-dependent phenotype of the *shibire Drosophila* mutant of dynamin [18]. This is suggestive of a role for dynamin in shaping the recycling vesicle membrane accompanied by a defect in the GTPase scission activity produced by the dynamin inhibitor [55], [56], [57].

In close agreement with studies based on the use of another dynamin inhibitor, dynasore, we found that dyngo-4a had a limited but significant effect on phasic quantal neurotransmitter release at low frequency stimulation as previously described [31]. This fits well with the FM1-43 destaining data generated by treatment of hippocampal neurons and sympathetic neurons with dynasore [14], [58]. At higher frequency stimulation, dyngo-4a did not significantly affect the rate of synaptic depression. Two possible scenarios can account for this lack of effect: (i) a dyngo-4a-insensitive endocytosis underpins fast recycling of synaptic vesicles or (ii) dyngo-4a-mediated inhibition of endocytosis taking place during synaptic depletion does not have time to generate fully functional synaptic vesicles and therefore does not significantly impact on the rate of synaptic depression. The latter scenario is more likely as dynasore has been shown to affect all forms of synaptic vesicle endocytosis [14]. Importantly, the lack of effect on synaptic depression is in good agreement with evidence from cortical neurons from dynamin-1 knockout mice [19].

In summary, our study provides evidence demonstrating that both actin and dynamin are differentially required for the development and maturation of activity-dependent bulk endocytosis. We show that bulk endocytosis is initiated during stimulation but the maturation process only occurs after the end of the stimulation. We propose that it may contribute to a mechanism by which nerve terminals couple exocytosis and bulk endocytosis, by sensing how much presynaptic membrane to retrieve in response to sustained stimulation. We also demonstrate that these dynamic presynaptic events are functionally linked to the recovery of synaptic transmission following synaptic depression.

## Materials and Methods

### Reagents and drugs

FM1-43 was purchased from Biotium (Hayward, CA). Dynasore, D-tubocurarine and chemicals for solutions were all purchased from Sigma (Sydney, Australia). For the synthesis of dyngo-4a (*3-hydroxynaphthalene-2-carboxylic acid (2,4,5-trihydroxybenzylidene) hydrazide*), 3-hydroxy-2- naphthoic hydrazide (0.2022 g, 0.1 mmol) and 2,4,5-trihydroxybenzaldehyde (0.1540 g, 1 mmol) were mixed with ethanol (25 mL) in a round-bottomed flask. The resultant mixture was set to reflux for 2 hrs, then allowed to cool and the solvent removed *in vacuuo*. The product (0.162 g, 45%) was recrystallized from ethanol. ^1^H and ^13^C spectra were recorded on a Bruker Advance AMX 300 MHz spectrometer at 300.13 and 75.48 MHz, respectively. Microanalyses were performed at MicroAnalytical Unit, Research School of Chemistry, at The Australian National University, Canberra.

### Tissue preparation

Electrophysiology and FM1-43 experiments were carried out on NMJ preparations of adult toads (*Bufo marinus*) with approval from the University of Queensland Animal Ethics Committee. Toads were euthanized by double pithing. The *iliofibularis* muscles with their motor nerve were dissected and pinned out on a Sylgard-lined organ bath (3 mL capacity) containing normal frog Ringer's solution (NFR; in mM: 116 NaCl, 2 KCl, 1.8 CaCl_2_, 1 MgCl_2_, 5 HEPES-OH, pH 7.3).

### FM1-43 labeling and confocal microscopy of nerve terminals

NMJ preparations were stimulated at 20 Hz for 10 min. A pulse of FM1-43 (5 µM) was applied during the last 5 min of stimulation. For passive labeling of nerve terminals, 10 µM FM1-43 was added to the bath for 10 min. The preparations were washed extensively with Ca^2+^-free Ringer's solution. For dyngo-4a and cytochalasin-D treatment, NMJ preparations were pretreated with dyngo-4a (30 µM) or cytochalasin D (4 µM) for 40 min prior to stimulation, FM1-43 labeling, and washing as above, followed by replacement with fresh Ca^2+^-free Ringer's solution containing either dyngo-4a or cytochalasin D. Confocal imaging of nerve terminals was carried out using a Zeiss 510-Meta confocal microscope. Confocal Z-section images were analyzed using either Zen (Zeiss, Oberkochen, Germany) or Imaris software (Bitplane, MN). Fluorescence recovery after photobleaching (FRAP) experiments were carried as previously described [25].

### Photoconversion

Our photoconversion protocol was adapted from previous studies (Harata et al., 2001; Meunier et al., 2010; Richards et al., 2003). AM1-43 labeled preparations were fixed in PBS containing 2% glutaraldehyde for 2 h. Muscles were then quickly washed twice in PBS, quenched in Phosphate Buffered Saline (PBS) containing 100 mM glycine pH 7.4 for 1 h, soaked in PBS containing 100 mM ammonium chloride for 5 min and further washed in PBS for 30 min. Samples were treated with 1.5 mg/ml 3, 3′-Diaminobenzidine (DAB) diluted in PBS for 30 min in the dark. The preparation was then illuminated under UV light through a 10× air objective (Carl Zeiss, Oberkochen, Germany) from a 100 W mercury lamp for 45–70 min depending on the rate of formation of the brown precipitate.

### Electron microscopy

Muscle preparations were pre-incubated with horseradish peroxidase (HRP, 10 mg/mL) for 5 hr. The preparations were then stimulated at 20 Hz for 10 min, followed by fixation in 2.5% glutaraldehyde, post-fixation in 1% osmium tetroxide, ethanol dehydration and Epon resin embedding (ProSciTech, Australia). Cross sections (60 nm thick) were stained with uranyl acetate and Reynolds lead citrate. NMJs were examined using a JEOL 1010 transmission electron microscope.

### Electrophysiological recordings

NMJ preparations were treated with d-tubocurarine (3–10 µM) for a period of 1–2 h to reduce the amplitude of EPPs and suppress the generation of action potentials and muscle contraction. Cytochalasin D (4 µM) was then added to the preparation for 40 min. EPPs were recorded at room temperature (18–20°C) with an intracellular glass capillary microelectrode filled with 2 M KCl (20–40 MΩ) using conventional intracellular recording techniques. The motor nerve of the isolated NMJ preparation was stimulated using silver chloride electrodes. Data were collected using Scope software (AD Instruments, CA) and stored in a Macintosh computer.

### Immunohistochemistry

Muscle preparations were fixed in 4% paraformaldehyde for 2 h at room temperature. Muscle fibers were teased apart and blocked in blocking buffer (3% normal horse serum, 0.5% bovine serum albumin (BSA), and 0.1% Triton X-100). Muscle fibers were then incubated overnight at 4°C with dynamin-1 primary antibody and AlexaFluor 555-conjugated α-bungarotoxin diluted in blocking buffer. After washing with phosphate-buffered saline (PBS), muscle fibres were incubated with Alexa 488-conjugated secondary antibody (Invitrogen), washed with PBS, and mounted (Prolong Gold; Invitrogen). Images were examined by confocal microscopy (LSM 510 Meta; Carl Zeiss, Jena, Germany). Quantification of the degree of colocalization was carried out using the color range tool in the LSM510 software.

### Morphometric analysis

In order to compare the amount of plasma membrane lost during the collapsing phase with the amount of internalized membrane generated in bulk endosomes and associated recycling vesicles, we needed to estimate (i) the changes in surface area of the plasma membrane occurring in the time-lapse imaging of motor nerve terminals and (ii) the membrane surface internalized in both bulk endosomes and surrounding recycling vesicles by morphometrical analysis.

#### Estimation of plasma membrane surface from confocal time-lapse imaging

We defined the region of interest (ROI) located mainly in the distal part of the motor nerve terminal to avoid possible contamination from the proximal axon. We used a combination of filters (Gaussian and thresholding filters) to eliminate unwanted background and a self-coded edge detection algorithm (Labview, National Instruments, Austin, TX) to obtain the edge coordinates of the nerve terminal within the ROI. Amphibian nerve terminals were roughly cylindrical in shape within the ROI. We therefore assumed that the volume of the nerve terminal within the ROI could be approximated as a series of very thin (one pixel thick) cylinders. The nerve terminal plasma membrane surface area 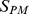 at time *t* was then calculated using (1):
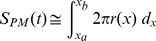
(1)

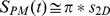
With 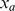 and 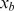 respectively, the start and the end pixels of the ROI along the nerve axis, 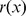 the radius of the nerve terminal in slice 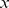, and 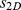 the surface area covered by the nerve terminal in the ROI from the confocal image. We reiterated the calculation using all the movie frames to obtain the dynamic changes of the plasma membrane surface area.

#### Estimation of membrane surface area internalized in bulk endosomes and associated recycling vesicles

From the confocal images, we counted the number of recyclosomes formed within our ROI, and for each, we measured the bulk endosome radius and the fluorescence halo outer radius using Laserpix (BioRad, Hercules, CA).

- Bulk endosome membrane surface area: The surface area 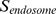 of a single endosome 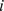 was calculated using (2):
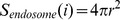
(2)


#### Membrane surface area associated with recycling vesicles surrounding bulk endosomes 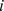


In order to retrieve the surface area of recycling vesicles, we determined the number of vesicles that were located in the halo. We first determined the volumetric density of recycling photoconverted vesicles from electron microscopy images (n = 4). The mean diameter of the photoconverted vesicles located within 350 nm of the bulk endosome was determined and the volumetric density of vesicles was calculated using (3):
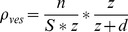
(3)where 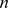 is the number of vesicles counted on the electron micrograph in a given surface area 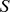 taken randomly within 350 nm of the limiting area of the bulk endosome, and 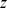 is the thickness of the electron microscope slice. The second term of the equation represents the probability that the counted vesicle lies completely within the slice. This correcting factor is important as some vesicles detected on electron micrographs were only partially located within the volume studied. We could then calculate the surface area of the recycling vesicles surrounding the bulk endosome with (4):
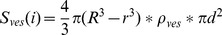
(4)where the first term of the equation is the volume of the fluorescence halo, and the last is the surface area of a single vesicle.

We then calculated the total internalized membrane surface area 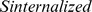 by summing the membrane surface area of all the 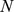 bulk endosomes and that of surrounding recycling vesicles detected in the ROI:
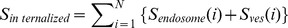
(5)


## Supporting Information

Figure S1 **Passive FM1-43 labeling was used to monitor maturation of bulk endocytosis at amphibian nerve terminals.** NMJ preparations were passively labeled by a 5 min pulse of FM1-43 (10 µM), followed by extensive washing with Ringer's solution. Electrical stimulation was elicited at 20 Hz for 10 min and visualized by time-lapse imaging starting from the beginning of the stimulation period. Recyclosomes are indicated by white arrows. Scale bar 5 µm.
(TIF)

Figure S2 **FM1-43 uptake into bulk endosomes is activity-dependent.** Motor nerve terminals were electrically stimulated as indicated in the figure. During the last 5 min of stimulation, FM1-43 (5 µM) was added to the preparation, which was subsequently washed several times in Ringer's solution containing d-tubocurarine (10–50 µM), after which the living motor nerve terminals were imaged by confocal microscopy. (**A**) In unstimulated nerve endings, FM1-43 staining was mainly localized on the plasma membrane. (**B**) At 20 Hz, most of the FM1-43 staining was closely associated with bulk endosomes. Scale bars 5 µm.
(TIF)

Figure S3 **FM-43-positve donut-like structures are not connected to the plasma membrane.** Fluorescence recovery after photobleaching (FRAP) analysis was performed on either unstimulated (**A**) or 20 Hz-stimulated (**B**) FM1-43-labeled motor nerve terminals. During the last 5 min of stimulation, FM1-43 (5 µM) was added, followed by extensive washing in Ringer's solution in the presence of D-tubocurarine (10–50 µM) to avoid muscular movements. FM1-43-stained nerve terminals were imaged before, during and after photobleaching of a defined region outlined in dotted circles. (**A**) Photobleaching of plasma membrane-embedded FM1-43 in unstimulated nerve terminals showed an almost immediate and full recovery. (**B**) In contrast, FM1-43 internalized in bulk endosomes and surrounding recycling vesicles exhibited minimal recovery after photobleaching. The calculated FRAP parameters of T_1/2_ (halftime of recovery), F_i_ and F_m_ (immobile and mobile fractions respectively) are indicated in the figure.
(TIF)

Figure S4 **Nerve terminal membrane surface retrieved during collapse correlates with that generated in bulk endosomes and associated recycling vesicles.** (**A**) Nerve terminals can be locally decomposed as a succession of very thin cylinders with a length d_x_ and radius r depending of the position along the nerve terminal. Integrating the plasma membrane surface over the whole nerve terminal provides a very good approximation of the amount of plasma membrane of the nerve terminal. (**B**) Representation of a transverse view of an electron micrograph slice. Vesicles partially located in adjoining slices (black) to the slice of thickness z were accounted for prior to obtaining the density of vesicles actually lying in the slice (yellow). (**C**) Representation of a recyclosome. The bulk endosome has a radius r, whereas the halo of associated recycling vesicles has a radius R.
(TIF)

Figure S5 **Dynamin inhibitors dynasore and MitMAB also block recyclosome formation and promote membrane tabulation.** (**A**) 20 Hz-stimulated nerve terminals treated with 100 µM dynasore and α-bungarotoxin (10 µM) displayed similar defects on bulk membrane retrieval characterized by appearance of dense FM1-43-labeled structures (arrow) located in close proximity to the plasma membrane (arrowhead) of the nerve terminal. (**B–D**) 20 Hz-stimulated NMJ preparation treated with MitMAB (30 µM) and α-bungarotoxin (10 µM), exhibit a block of endocytosis accompanied with an accumulation of FM1-43 on the presynaptic plasma membrane (closed arrow) and in internal tubulation (arrowhead). An accumulation of the styryl dye fluorescence in striated pattern of the plasma membrane could be observed (**D**, open arrow). Bars 5 µm.
(TIF)

Figure S6 **Ultrastructural analysis of nerve terminals shows that dyngo-4a promotes the formation of malformed and tubular membrane structures.** NMJ preparations were stimulated at 20 Hz for 10 min either in the absence (**A**) or presence (**B–G**) of dyngo-4a. Untreated nerve terminals displayed regularly shaped bulk endosomes (**A**, black arrows). In contrast, nerve terminals treated with dyngo-4a displayed malformed (**E**) and tubular (**B–D** and **F–G**, black arrowheads) membrane structures. In some instances, long tubular structures could be observed originating from the plasma membrane and elongating into the intraterminal space (**C**, black arrowheads).
(TIF)

Figure S7 **Dyngo-4a has a limited but significant effect on low-frequency quantal phasic neurotransmitter release at the neuromuscular junction.** (**A** and **B**), NMJ preparations were stimulated at 0.5 Hz to induce evoked phasic neurotransmitter release either in the absence (black) or presence (red) of dyngo-4a (30 µM) and EPPs were recorded for a period of 3 min. Time-course of EPP amplitude (normalized to the initial EPP amplitude) elicited at 1 Hz in control untreated (**C** and **D**) or dyngo-4a-treated nerve terminals (100 µM) (**E** and **F**) over a period of 30 min. The insets in (**C** and **E**) show representative EPP traces at the indicated time points. (**D** and **F**), Comparison of the EPP averaged amplitudes in the indicated conditions, (n = 3). Data shown as mean ± S.E.M and statistic significance was determined using Student's *t* test.
(TIF)

Movie S1 **Time-lapse imaging of nerve terminal morphological changes.** The NMJ preparation was pre-labeled with FM1-43 by electrical stimulation (2 Hz for 5 min), and extensively washed in normal Ringer's solution. Nerve terminals were then stimulated at 20 Hz for 10 min and visualized by time-lapse confocal imaging starting from the final 4 min of stimulation. The asterisk denotes the end of the stimulation, after which the nerve terminal undergoes various remodeling stages: the progressive increase in plasma membrane diameter (bulging), the rapid increase in intraterminal fluorescence following the collapsing in plasma membrane diameter, and the appearance of the ‘bulk endosomes’.
(MPG)

Movie S2 **Cytochalasin D inhibits presynaptic membrane bulging and bulk endocytosis.** The NMJ preparation was pre-treated with 4 µM cytochalasin D for 40 min followed by prelabeling with 10 µM FM1-43 for 5 min. The preparation was extensively washed in normal Ringer's solution prior to stimulation at 20 Hz for 10 min. Confocal time-lapse imaging was started immediately after the end of stimulation.
(MPG)

Movie S3 **Dynamin inhibition results in formation of plasma membrane tubular structures.** NMJ preparations were pre-treated with 30 µM dyngo-4a for 40 min followed by prelabeling with 10 µM FM1-43 for 5 min. The preparation was extensively washed in normal Ringer's solution prior to stimulation at 20 Hz for 10 min. Confocal Z-stack imaging revealed numerous FM1-43-positive tubular membrane structures. 3-dimensional modeling of the tubular structures was performed using Imaris imaging software.
(M4V)
