# Supplementary figures and images for: Actin- and Dynamin-Dependent Maturation of Bulk Endocytosis Restores Neurotransmission following Synaptic Depletion
Source: PLoS One. 2012 May 22;7(5):e36913. doi: 10.1371/journal.pone.0036913 (PMC3358275; doi:10.1371/journal.pone.0036913)

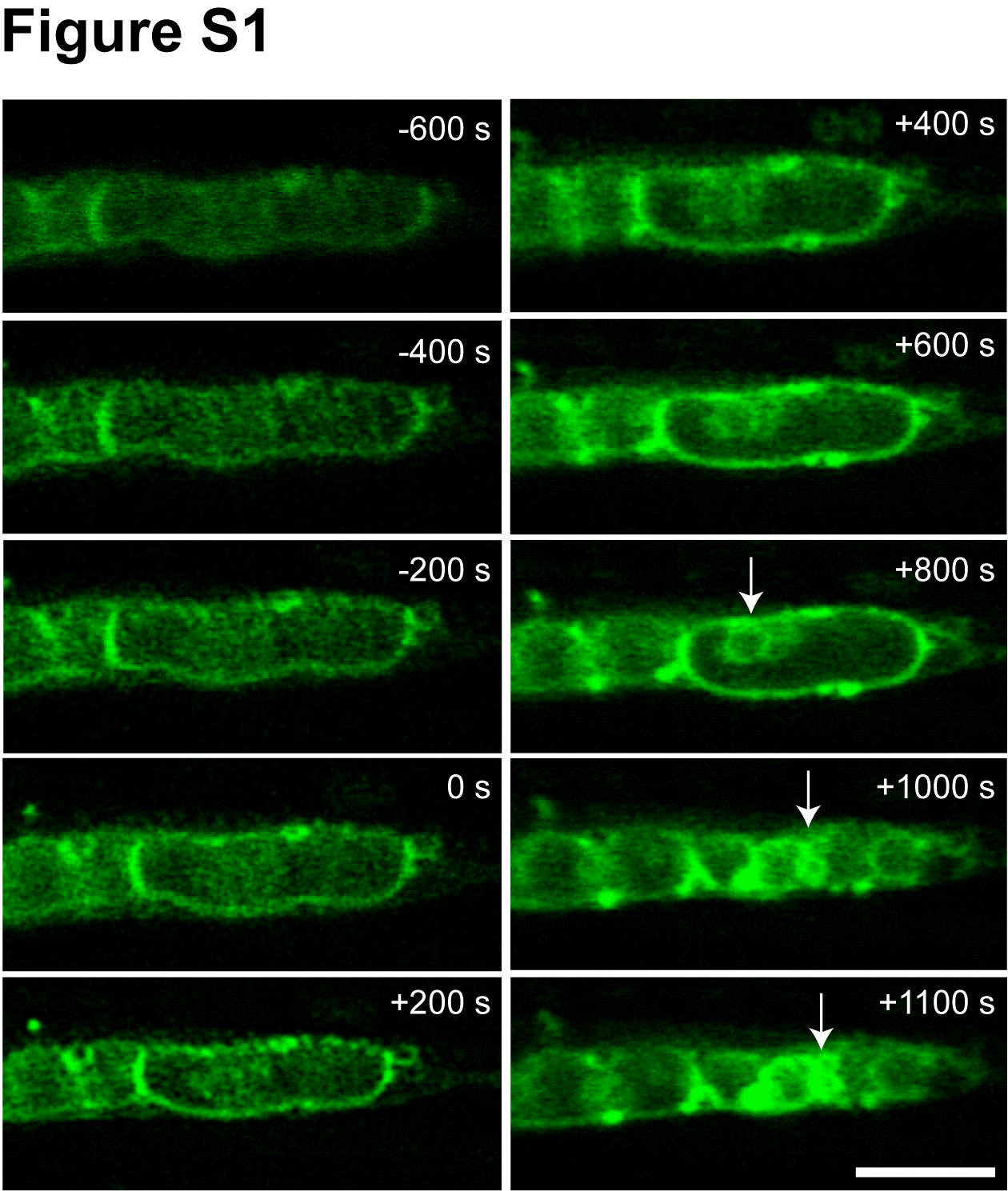

Supplement: Figure S1 — Passive FM1-43 labeling was used to monitor maturation of bulk endocytosis at amphibian nerve terminals. NMJ preparations were passively labeled by a 5 min pulse of FM1-43 (10 µM), followed by extensive washing with Ringer's solution. Electrical stimulation was elicited at 20 Hz for 10 min and visualized by time-lapse imaging starting from the beginning of the stimulation period. Recyclosomes are indicated by white arrows. Scale bar 5 µm. (TIF) [file pone.0036913.s001.tif]

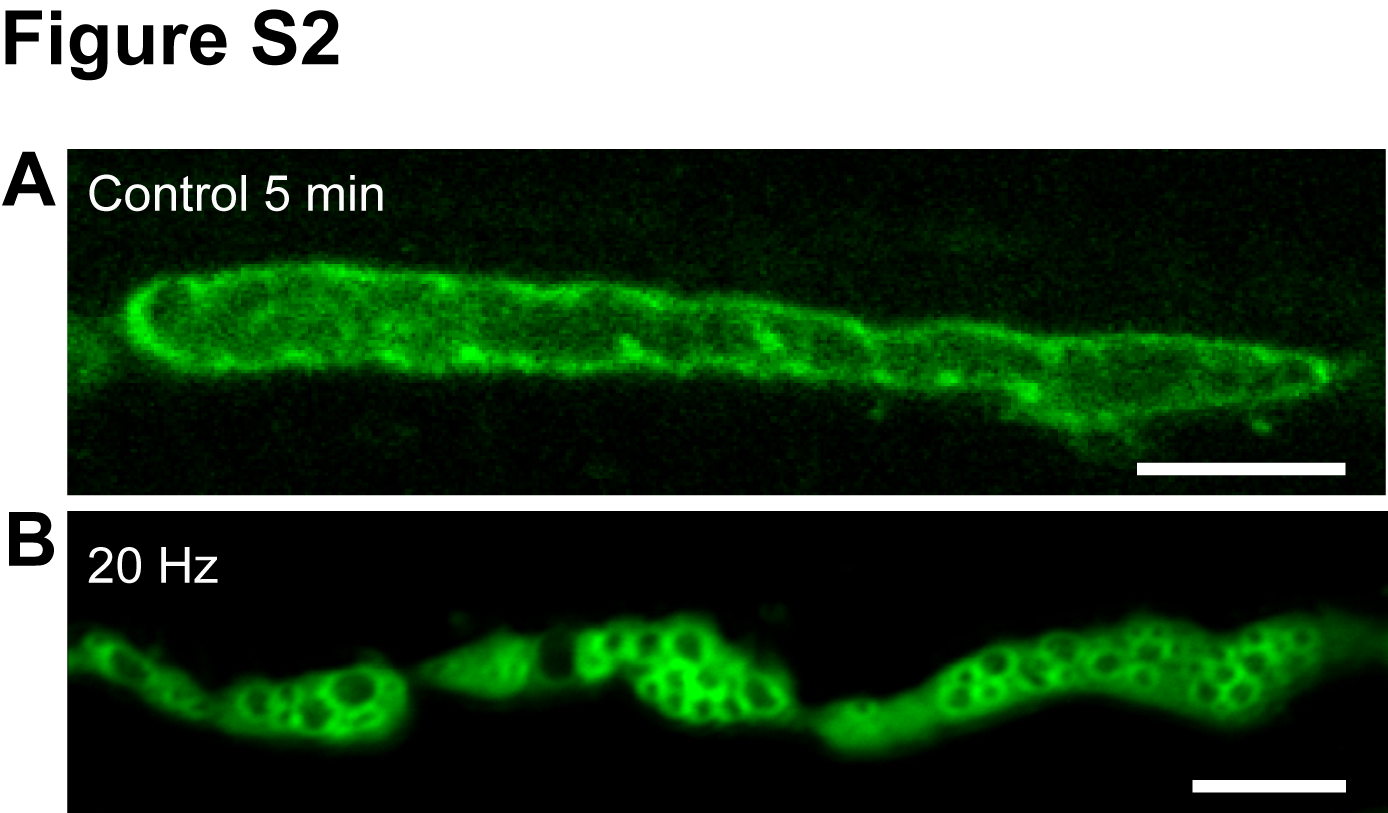

Supplement: Figure S2 — FM1-43 uptake into bulk endosomes is activity-dependent. Motor nerve terminals were electrically stimulated as indicated in the figure. During the last 5 min of stimulation, FM1-43 (5 µM) was added to the preparation, which was subsequently washed several times in Ringer's solution containing d-tubocurarine (10–50 µM), after which the living motor nerve terminals were imaged by confocal microscopy. (A) In unstimulated nerve endings, FM1-43 staining was mainly localized on the plasma membrane. (B) At 20 Hz, most of the FM1-43 staining was closely associated with bulk endosomes. Scale bars 5 µm. (TIF) [file pone.0036913.s002.tif]

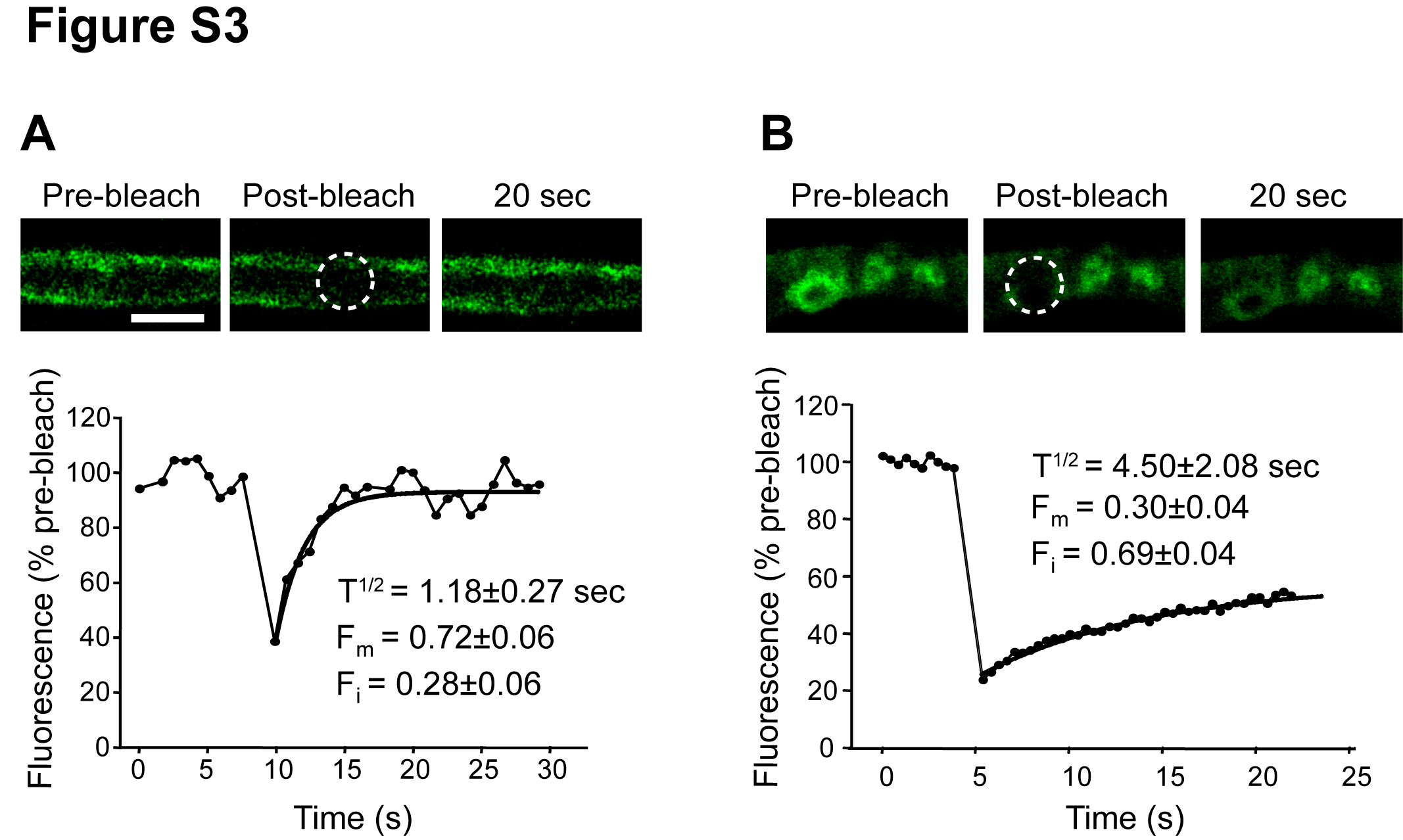

Supplement: Figure S3 — FM-43-positve donut-like structures are not connected to the plasma membrane. Fluorescence recovery after photobleaching (FRAP) analysis was performed on either unstimulated (A) or 20 Hz-stimulated (B) FM1-43-labeled motor nerve terminals. During the last 5 min of stimulation, FM1-43 (5 µM) was added, followed by extensive washing in Ringer's solution in the presence of D-tubocurarine (10–50 µM) to avoid muscular movements. FM1-43-stained nerve terminals were imaged before, during and after photobleaching of a defined region outlined in dotted circles. (A) Photobleaching of plasma membrane-embedded FM1-43 in unstimulated nerve terminals showed an almost immediate and full recovery. (B) In contrast, FM1-43 internalized in bulk endosomes and surrounding recycling vesicles exhibited minimal recovery after photobleaching. The calculated FRAP parameters of T1/2 (halftime of recovery), Fi and Fm (immobile and mobile fractions respectively) are indicated in the figure. (TIF) [file pone.0036913.s003.tif]

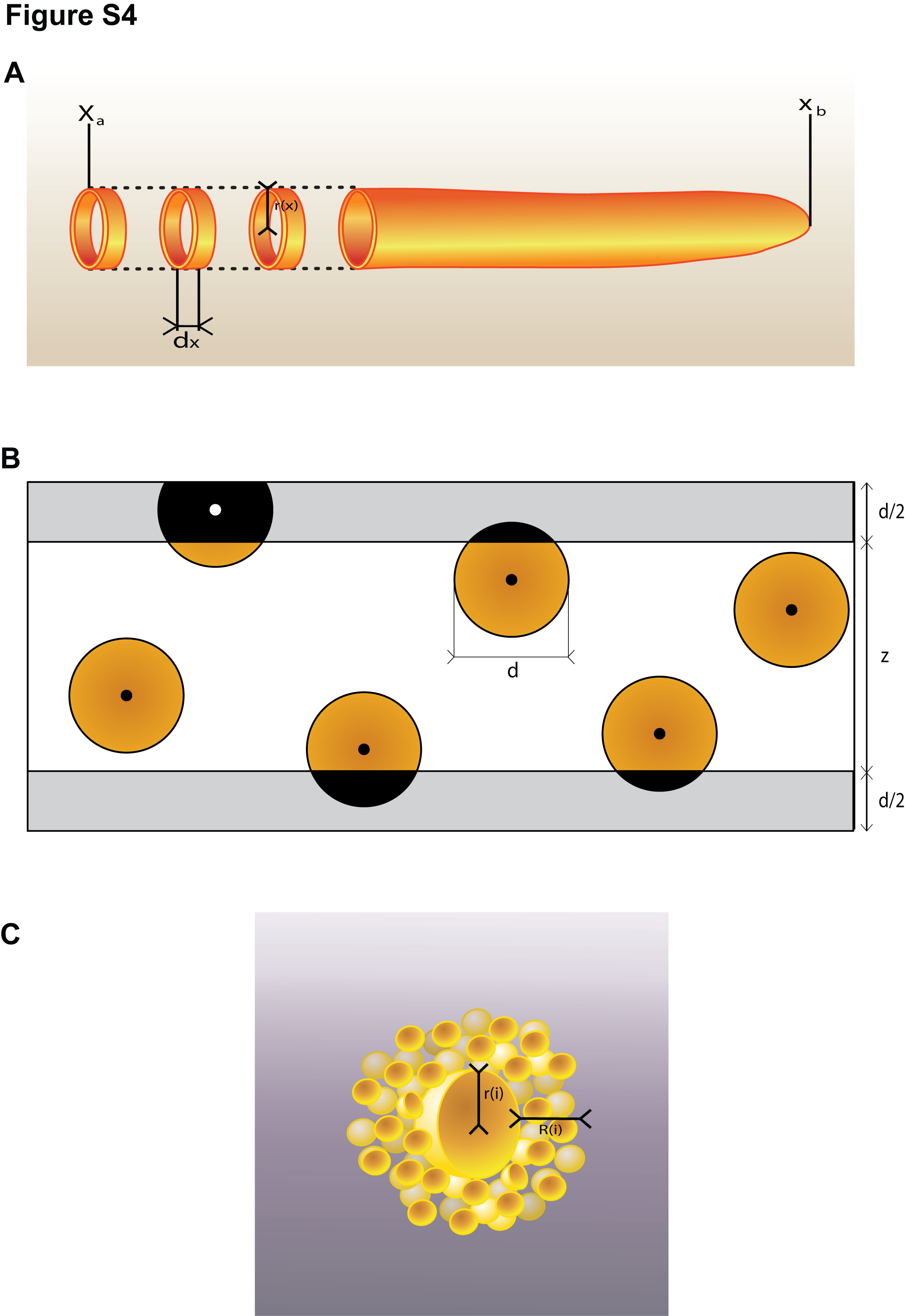

Supplement: Figure S4 — Nerve terminal membrane surface retrieved during collapse correlates with that generated in bulk endosomes and associated recycling vesicles. (A) Nerve terminals can be locally decomposed as a succession of very thin cylinders with a length dx and radius r depending of the position along the nerve terminal. Integrating the plasma membrane surface over the whole nerve terminal provides a very good approximation of the amount of plasma membrane of the nerve terminal. (B) Representation of a transverse view of an electron micrograph slice. Vesicles partially located in adjoining slices (black) to the slice of thickness z were accounted for prior to obtaining the density of vesicles actually lying in the slice (yellow). (C) Representation of a recyclosome. The bulk endosome has a radius r, whereas the halo of associated recycling vesicles has a radius R. (TIF) [file pone.0036913.s004.tif]

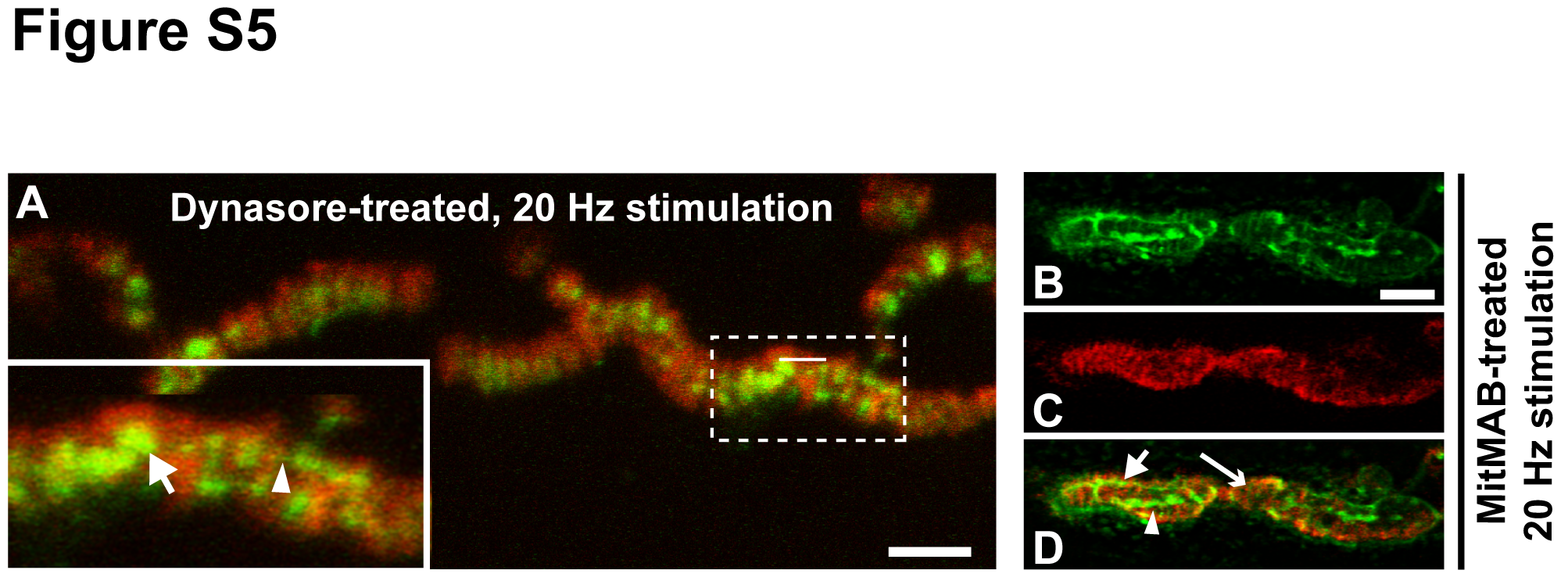

Supplement: Figure S5 — Dynamin inhibitors dynasore and MitMAB also block recyclosome formation and promote membrane tabulation. (A) 20 Hz-stimulated nerve terminals treated with 100 µM dynasore and α-bungarotoxin (10 µM) displayed similar defects on bulk membrane retrieval characterized by appearance of dense FM1-43-labeled structures (arrow) located in close proximity to the plasma membrane (arrowhead) of the nerve terminal. (B–D) 20 Hz-stimulated NMJ preparation treated with MitMAB (30 µM) and α-bungarotoxin (10 µM), exhibit a block of endocytosis accompanied with an accumulation of FM1-43 on the presynaptic plasma membrane (closed arrow) and in internal tubulation (arrowhead). An accumulation of the styryl dye fluorescence in striated pattern of the plasma membrane could be observed (D, open arrow). Bars 5 µm. (TIF) [file pone.0036913.s005.tif]

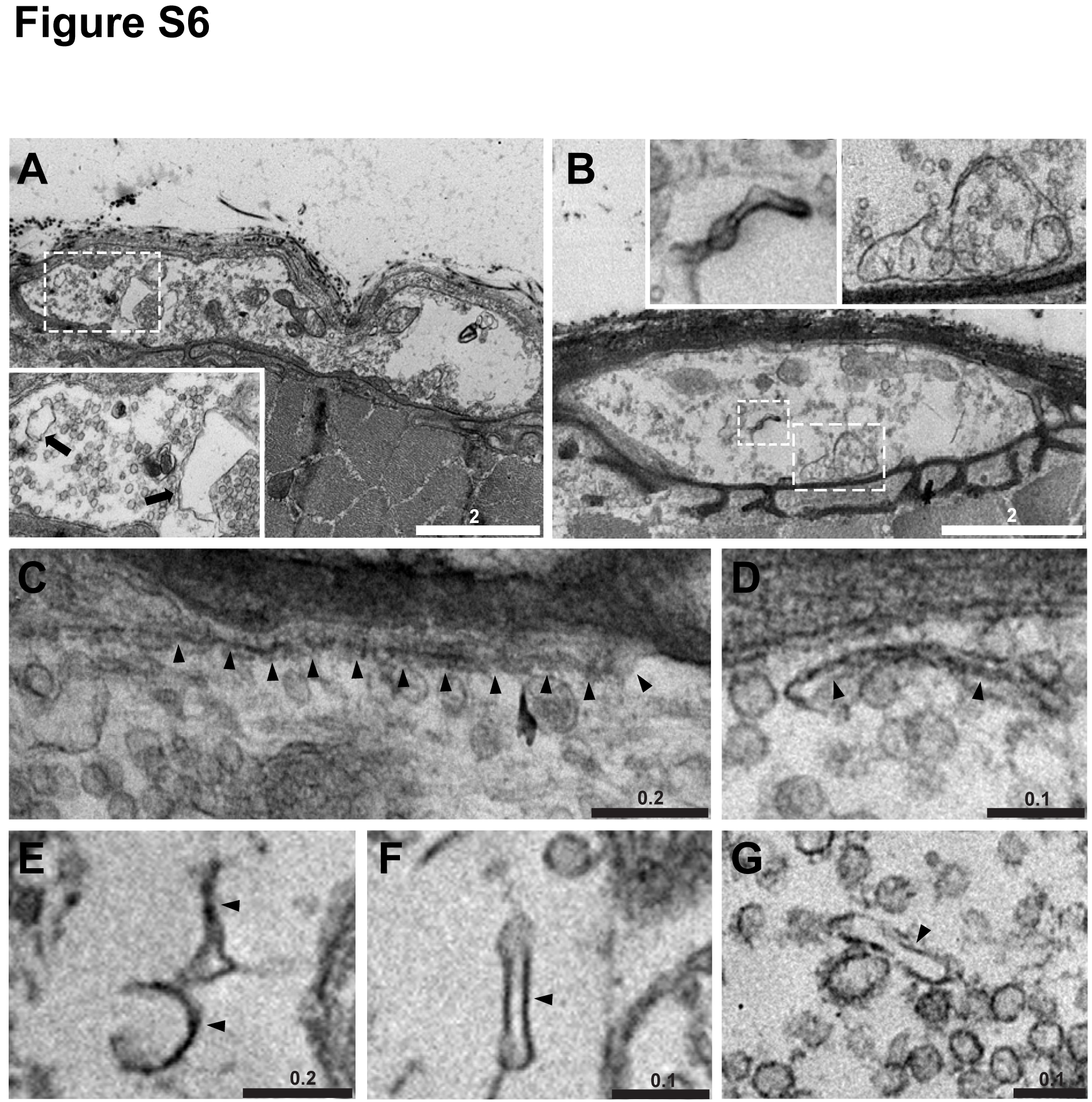

Supplement: Figure S6 — Ultrastructural analysis of nerve terminals shows that dyngo-4a promotes the formation of malformed and tubular membrane structures. NMJ preparations were stimulated at 20 Hz for 10 min either in the absence (A) or presence (B–G) of dyngo-4a. Untreated nerve terminals displayed regularly shaped bulk endosomes (A, black arrows). In contrast, nerve terminals treated with dyngo-4a displayed malformed (E) and tubular (B–D and F–G, black arrowheads) membrane structures. In some instances, long tubular structures could be observed originating from the plasma membrane and elongating into the intraterminal space (C, black arrowheads). (TIF) [file pone.0036913.s006.tif]

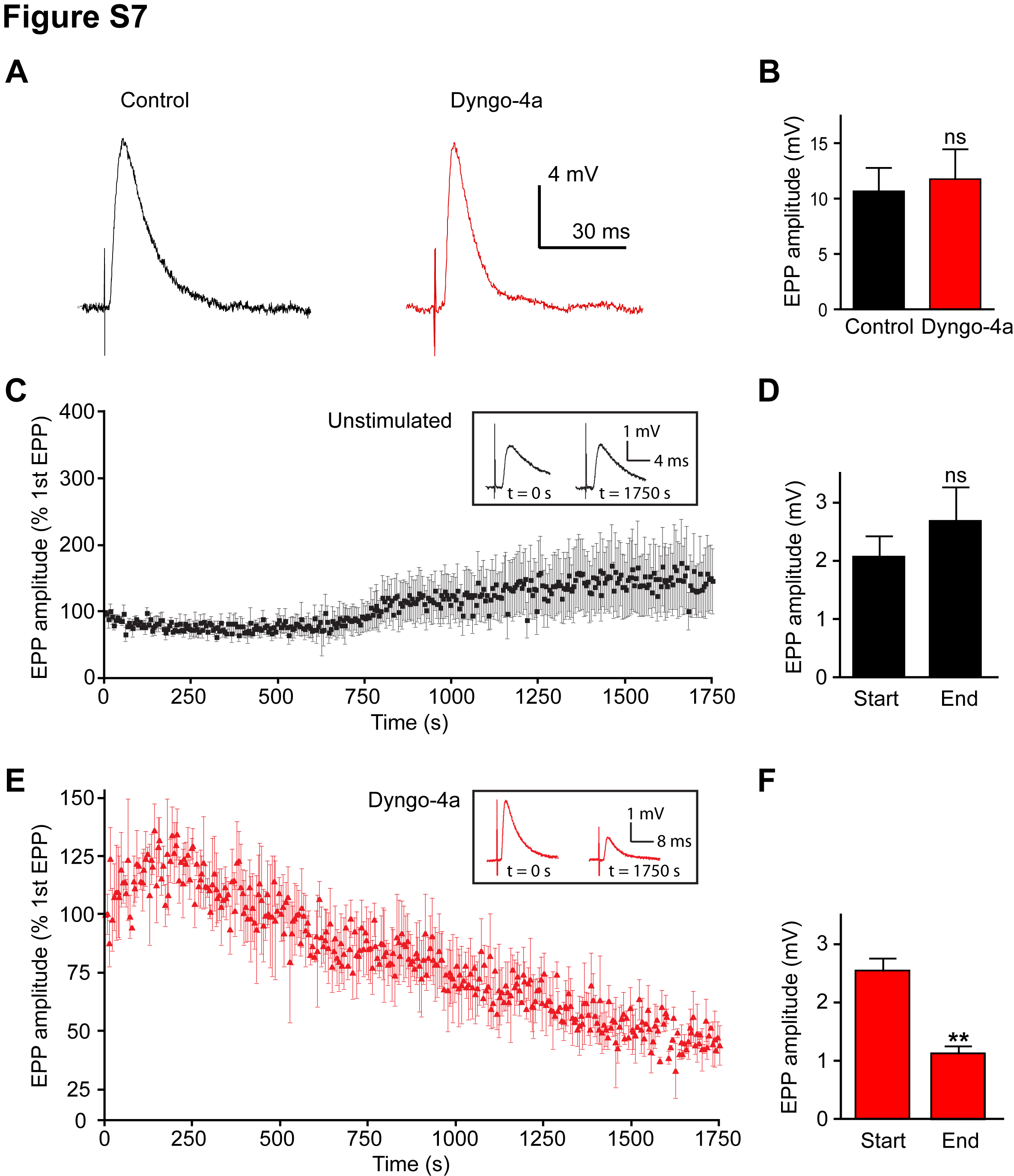

Supplement: Figure S7 — Dyngo-4a has a limited but significant effect on low-frequency quantal phasic neurotransmitter release at the neuromuscular junction. (A and B), NMJ preparations were stimulated at 0.5 Hz to induce evoked phasic neurotransmitter release either in the absence (black) or presence (red) of dyngo-4a (30 µM) and EPPs were recorded for a period of 3 min. Time-course of EPP amplitude (normalized to the initial EPP amplitude) elicited at 1 Hz in control untreated (C and D) or dyngo-4a-treated nerve terminals (100 µM) (E and F) over a period of 30 min. The insets in (C and E) show representative EPP traces at the indicated time points. (D and F), Comparison of the EPP averaged amplitudes in the indicated conditions, (n = 3). Data shown as mean ± S.E.M and statistic significance was determined using Student's t test. (TIF) [file pone.0036913.s007.tif]
